# Advances in the mechanism for steroid-induced osteonecrosis of the femoral head

**DOI:** 10.1038/s41413-025-00477-2

**Published:** 2026-02-12

**Authors:** Runze Zhou, Yixin Bian, Xuejie Cai, Hanyang Sun, Zehui Lv, Yiming Xu, Yingjie Wang, Han Wang, Wei Zhu, Bin Feng, Xisheng Weng

**Affiliations:** Department of Orthopedic Surgery, State Key Laboratory of Complex Severe and Rare Diseases, Peking Union Medical College Hospital, Chinese Academy of Medical Science and Peking Union Medical College, Beijing, China

**Keywords:** Bone, Bone quality and biomechanics

## Abstract

Steroid-induced osteonecrosis of the femoral head (SONFH) is a debilitating condition resulting from the use of glucocorticoids, commonly prescribed for immune-related and inflammatory diseases. Understanding the mechanisms driving SONFH remains a significant challenge, complicating efforts to prevent and treat the condition. While genetic predispositions, impaired blood supply, and metabolic changes are recognized contributors, the complex interplay between these factors is not yet fully understood. Recent research has shed light on the pathogenesis of SONFH, exploring it from multiple perspectives, including tissue-level damage, cellular dysfunction, and molecular pathways. This review summarizes these recent advancements, providing an integrated understanding of the onset and progression of the condition. Additionally, it highlights emerging therapeutic strategies that potentially pave the way for more effective treatments in the future.

## Introduction

Osteonecrosis of the femoral head (ONFH) is a progressive disease affecting primarily individuals aged 30–50, characterized by femoral head ischemia and osteocyte death.^1–3^ ONFH cases have generally increased over the past decade worldwide.^4,5^ Patients with ONFH often report pain localized to the hip, buttock, or groin, accompanied by restricted hip joint motion, with nearly 80% of patients eventually experiencing femoral head collapse^2,6–9^ Diagnostic imaging plays a crucial role in identifying the disease, with the “double-line sign” on magnetic resonance imaging (MRI) and the “crescent sign” on X-rays serving as characteristic features of ONFH.^2,9–11^ The main types of ONFH are categorized into generally traumatic and non-traumatic ONFH (caused by glucocorticoid use, alcohol abuse, or idiopathic).

Glucocorticoids (GCs) use is the most common non-traumatic cause of ONFH, which ranges about 40%–60% among all non-traumatic ONFH patients.^4,5^ A novel epidemiology showed a rise to 75% of non-traumatic ONFH related to GCs treatment.^4^ GCs exert their effects by binding to the glucocorticoid receptor (GR), that expressed in nearly all cells, regulating cell growth, differentiation, and apoptosis.^9,12^ In its inactive state, GR resides in the cytoplasm, associated with a complex of proteins such as heat shock proteins (HSP90, HSP70) and immunophilins (e.g., FKBP51, FKBP52). Upon GCs binding, the complex disassembles, activating GR, which then translocates to the nucleus, where it interacts with target genes, initiating transcriptional responses that mediate GCs' effects.^13–15^ GC-induced ONFH has been proven to vary from other types of ONFH in many aspects. Patients with SONFH tend to exhibit more rapid disease progression, higher rates of bilateral involvement, and earlier femoral head collapse compared to those with alcohol-induced or idiopathic ONFH.^16–18^ Notably, while both SONFH and alcohol-induced ONFH are characterized by lipid dysregulation, SONFH is marked by GC-induced bone marrow adipogenesis, whereas the alcohol-induced subtype is more closely related to hepatic lipid metabolism and dysfunction.^17,19^

**Fig. 1:**
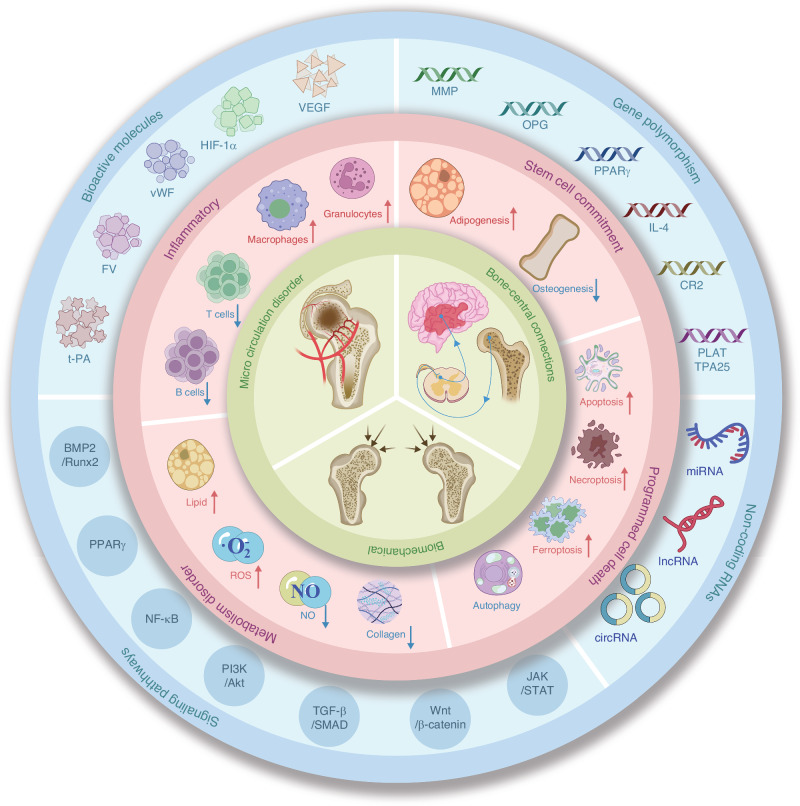
Potential mechanism of steroid-induced osteonecrosis of the femoral head

Advances in medical imaging and bioinformatics have enhanced our understanding of SONFH, offering novel insights into its pathophysiology.^20–22^ However, a comprehensive review of the underlying causes of SONFH has yet to be concluded. In this review, we highlight recent findings on the mechanisms of SONFH from multiple perspectives. General pathological changes, such as impaired blood supply and biomechanical alterations, are key contributors to the disease process. Abnormal neuronal transmission has also been identified as a factor for SONFH pathology. At the cellular level, irregular cell differentiation and increased cell death play critical roles in the progression of SONFH. On a molecular scale, altered activity of crucial factors and related signaling cascades, as well as disordered non-coding RNAs (ncRNAs), have been implicated in the pathogenesis of the disease. Finally, the role of genetic variations in SONFH susceptibility is increasingly being recognized (Fig. 1). By summarizing these advancements, we aim to provide a comprehensive understanding of SONFH mechanisms, which may inspire further research and develop novel treatment strategies.

## General pathological alternation

ONFH has been characterized by a series of general pathological changes. However, SONFH, even though it emphasizes more on the effect of GCs, shares several general pathological alternations with other ONFH. Typically, vascular occlusion leads to insufficient blood supply, causing osteonecrosis and biomechanical weakening, which often further alters the alignment of the lower limb axis. In addition, recent studies revealed that severe pain and disrupted bone homeostasis can be linked to irregular central nervous system-bone connections, highlighting the complex interplay between skeletal and nervous systems in the SONFH process. Studies investigating the general pathological changes in SONFH are listed in Table 1.

**Table 1:** Studies investigating the general pathological changes in SONFH

Reference	Macroscopic changes	Animals	Involved cells	Evaluation methodology	Outcome
Zheng et al.^24^	Microcirculation alternations	/	/	Histology	The collapse of ONFH initiated at the lateral column of the femoral head within the necrotic lesion, with the lateral column experiencing ischemia.
Kim, H. et al.^319^	Microcirculation alternations	/	/	Histology	The supply of nutrition for the proximal femoral growth plate did not depend only on the epiphyseal vasculature.
Li et al.^26^	Microcirculation alternations	/	/	Dynamic contrast-enhanced (DCE) MRI	Increased permeability of the vascular wall, blood stasis in the posterior circulation, high intraosseous pressure in the femoral head, and decreased arterial blood flow were the main features of ONFH.
Drescher, W. et al.^27^	Microcirculation alternations	Rats	/	Statistical analysis	Short-term treatment with high-dose methylprednisolone causes a reduction in osseous blood flow.
Wu et al.^28^	Microcirculation alternations	/	/	Proteomics analysis, immunohistochemical, tube morphology	S100A9 rendered avascular damage, eventually accelerating femoral head deterioration by reducing angiogenesis.
Yue et al.^52^	Microcirculation alternations	Rats	BMECs	qPCR, miRNA microarray analysis	SONFH might be induced by miRNA changes in BMECs.
Mao et al.^268^	Microcirculation alternations	/	BMECs	Immunofluorescence	CircCDR1as decreased angiogenesis and proliferation of BMECs by sponging miR-135b and upregulating FIH-1.
Lu et al.^137^	Microcirculation alternations	/	BMECs	miRNA microarray analysis, qPCR	GCs promoted BMECs to express vasoconstrictors and procoagulant factors along with their related receptors, while downregulating the expression of vasodilators and their receptors.
Chen et al.^58^	Microcirculation alternations	/	Early EPCs, endothelial colony-forming cells (ECFCs)	Flow Cytometry, immunofluorescence	Early EPCs and ECFCs were impaired in number and function, respectively, in SONFH.
Dimova, I. et al.^57^	Microcirculation alternations	Mice	BMSCs, EPCs	Immunohistochemical, adhesion assay	SDF-1/CXCR4 promoted angiogenesis and further highlighted the stabilizing role of BMDC in vascular remodeling.
Guo et al.^29^	Microcirculation alternations	/	HUVECs	Immunohistochemical, qRT-PCR	Vitamin B2 could promote the proliferation and migration of HUVECs and inhibit apoptosis through the PI3K/Akt signaling pathway.
Fraitzl, C. et al.^30^	Biomechanical alternations	/	/	X-ray	The mean α-angle for anteroposterior and lateral radiographs of ONFH patients were obviously larger than the control group.
Karasuyama et al.^31^	Biomechanical alternations	/	/	Finite element model (FEM), histology	Shear stress and shear strain tend to accumulate around thickened bone trabeculae located at the boundary.
Xing et al.^22^	Abnormal bone-central connection	/	/	Functional MRI (fMRI)	ONFH patients had abnormal activity of the brain network, especially in the sub-brain area.
Feng et al.^20^	Abnormal bone-central connection	/	/	Resting-state fMRI(rs-fMRI)	General ALFF differences were found between the patients and the healthy throughout the occipital, parietal, frontal, prefrontal, and temporal cortices.
Wang et al.^43^	Abnormal bone-central connection	Rabbits	/	Immunohistochemistry	ONFH was chronologically associated with changes in neural factors.
Chen et al.^45^	Abnormal bone-central connection	Mice	/	Behavioral analysis, ELISA, immunohistochemistry	PGE2 promoted skeletal regeneration through sensory nerves through the PGE2-EP4 pathway.
Hu et al.^46^	Abnormal bone-central connection	Mice	BMSCs	Representative μCT, immunohistochemistry	PGE2/EP4 sensory nerve pathway could regulate BMSCs differentiation to osteoblasts.

### Microcirculation disorder

**Fig. 2:**
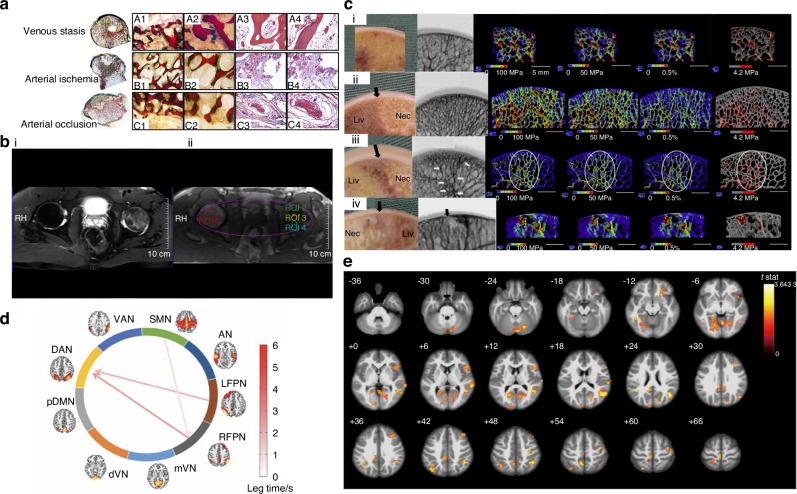
General pathological alternations in SONFH. **a** Results of the pathological sections of osteonecrotic femoral heads focusing on various blood supply changes in the lateral weight-bearing area. (A1, B1, and C1) Detailed results are shown in A2, B2, and C2. A3,4, B3,4, C3,4 showed the HE staining conditions. Reproduced with permission. Copyright 2022, The Journal of Bone and Joint Surgery.^24^ **b** T2WI lipid suppression image demonstrating necrosis and edema of the left femoral head. (i) Different Regions of interest (ROIs) selected by Tissue 4D processing software (ROI-1, red circle, healthy femoral heads; ROI-2, green circle, necrotic area; ROI-3, yellow circle, boundary area; ROI-4, blue circle, edema area). (ii) Reproduced with permission. Copyright 2023, Li et al.^26^ **c** Macroscopic appearance, specimen radiograph, von Mises equivalent stress, octahedral shear stress, octahedral shear strain, and fracture analysis at the normal area(i), Type1 (ii), 2 (iii), and 3 (iv) ONFH. Reproduced with permission. Copyright 2015, Elsevier.^31^ **d** The functional network connectivity (FNC) of the brain differed in patients with ONFH compared to healthy controls, specifically in the sub-brain network. Reproduced with permission. Copyright 2022, Pain Physician.^22^ **e** *T*-test statistical difference maps between the ONFH patients and healthy controls. Areas with different activity were highlighted which mainly included right middle occipital gyrus (MOG), right inferior parietal lobule, left angular gyri, right insula, right superior temporal gyrus, right lingual gyrus, left median cingulate and paracingulate gyri, left precuneus, left cuneus, right parahippocampal gyrus (PHG) right middle temporal gyrus, the left calcarine fissure, both sides of the precentral gyrus (PreCG), the bilateral postcentral gyrus (PoCG), the right triangular part of the inferior frontal gyrus (IFGtri), the right middle frontal gyrus, and the right paracingulate gyri. Reproduced with permission. Copyright 2022, Feng et al.^20^

The main nutrient source of the femoral head is the medial femoral circumflex artery (MFCA).^23^ Occlusion of its lateral epiphyseal branches, particularly the superior retinacular artery, is critical for femoral head necrosis and collapse, whereas medial branches are less involved.^24^ This occlusion is always attributed to venous stasis, arterial ischemia, or arterial occlusion (Fig. 2a). In addition, occlusion of the artery of the ligamentum teres, a branch of the obturator artery, is reported to be another common risk factor associated with ONFH.^25^

The microcirculatory changes in morphology and perfusion caused by GC treatment have been extensively studied. Li et al.^26^ used dynamic contrast-enhanced magnetic resonance imaging (DCE-MRI) to evaluate vessel morphology and perfusion parameters in both healthy and lesion areas of the femoral head, which showed an increase in vessel wall permeability and extravascular space, alongside a decrease in posterior circulation function, potentially explaining the elevated intraosseous pressure observed in patients with SONFH (Fig. 2b). Similarly, Drescher et al.^27^ demonstrated that brief exposure to high-dose methylprednisolone significantly reduced bone blood flow and potentially triggered early stages of SONFH. Apart from poor perfusion, GC treatment also inhibits angiogenesis. Histopathological analysis of osteonecrotic femoral head tissue reveals a hypovascular condition compared to femoral head fracture specimens.^28^ Interestingly, vitamin B2 has shown potential in mitigating the effects of SONFH by promoting blood vessel regeneration, which facilitates the migration of human umbilical vein endothelial cells (HUVECs) and contributes to increased bone mass.^29^

### Biomechanical alternations

The compromised vascular system and subsequent necrosis result in uneven load distribution on the femoral head.^25^ This leads to progressive bone damage and even altered lower limb alignment. Research indicates that patients with SONFH exhibit significantly higher α-angles, which are associated with an increased risk of cam-type femoroacetabular impingement (FAI). This condition can repeatedly injure the superior retinacular vessels, exacerbating the disease.^30^ Finite element modeling (FEM) has identified concentrated mechanical stresses at the interface between necrotic and healthy bone, further accelerating femoral head collapse (Fig. 2c).^31^ Despite these biomechanical insights, no significant differences in trabecular properties or bone volume have been found between SONFH and osteoarthritis.^32^ Molecularly, PIEZO channels (PIEZO1/2) and transient receptor potential (TRP) channels are crucial mechanosensitive regulators, recognizing high-intensity mechanical input and low-intensity mechanical input, respectively.^33^ Activation of PIEZO1 and TRPV4 channels has been found to increase osteogenic gene expressions that respond to mechanical stress, providing a potential treatment target for SONFH.^34–36^

### Bone-central nervous system (CNS) communication

Recent studies have revealed that the CNS plays a critical role in regulating bone sensations and metabolism. Abnormal activity in the cerebral cortex has been linked to adverse outcomes in ONFH. Alterations in brain functional regions have been implicated in the dysfunction and pain associated with ONFH. A prior study identified abnormal activity within nine resting-state networks in ONFH patients compared to healthy controls. Increased functional connectivity was observed between the sensorimotor network and the right frontoparietal network, as well as between the dorsal attention network and the bilateral frontoparietal network, potentially contributing to functional impairments (Fig. 2d).^22^ Additionally, Feng et al.^20^ utilized resting-state functional MRI (rs-fMRI) to analyze the amplitude of low-frequency fluctuations (ALFF) in ONFH patients and found altered spatial patterns of brain activity, particularly in regions associated with sensorimotor functions, pain, emotion, and cognition (Fig. 2e).

CNS not only participates in pain perception but also in the regulation of bone metabolism. In general, increased sympathetic tone and decreased vagal tone are associated with reduced bone mass.^37^ Neuropeptides such as CGRP and VIP, released by peripheral nerves, promote osteoblast differentiation by enhancing bone morphogenetic protein 2 (BMP2) expression.^38–40^ In contrast, neuropeptide Y (NPY) and substance P (SP) promote bone resorption by stimulating osteoclast activity.^41,42^ Notably, altered expression levels of neural factors have been observed in rabbit models of GC-induced osteonecrosis, with downregulation of osteogenic neuropeptides (e.g., CGRP, VIP) and upregulation of those that favor bone resorption (e.g., NPY, SP).^43^

PGE2–EP4 signaling has been shown to inhibit sympathetic outflow by promoting phosphorylation of cAMP response element-binding protein (CREB) in the hypothalamus, thereby facilitating osteogenesis by directing mesenchymal stromal cell (MSC) differentiation toward the osteoblastic lineage.^44–46^ Additionally, PGE2 can suppress neuropeptide Y (NPY) expression in the arcuate nucleus, thereby modulating both lipid metabolism and osteogenic activity.^46,47^ In SONFH models, GCs have been reported to downregulate PGE2 expression, thereby disrupting skeletal homeostasis.^37^

Leptin, a molecule secreted by adipose tissue, is well known for its role in regulating bone formation through the hypothalamic–pituitary–gonadal (HPG) axis. Leptin increases circulating estrogen levels and acts within the hypothalamus to improve bone metabolism. By contrast, overactivation of the HPG axis can lead to stress-induced bone loss, suggesting a dose-dependent effect of leptin on bone homeostasis.^48^

Parathyroid hormone (PTH), a key regulator of bone remodeling whose pulsatile secretion is disrupted by GCs, has also been found to be under CNS control.^49^ Circulating PTH stimulates neurons in the subfornical organ (SFO), which project GABAergic signals to the paraventricular nucleus (PVN), forming a bidirectional feedback loop that modulates peripheral PTH levels. These findings underscore the broader role of the CNS in regulating bone remodeling, with potential relevance to the progression of SONFH.^50^

These findings underscore the broader role of the CNS in regulating bone mass and remodeling through both sensory input and central autonomic outputs, a mechanism particularly relevant to the development and progression of SONFH.

## Cell niche dysregulation

GCs disrupt cellular homeostasis, which plays a pivotal role in SONFH pathogenesis. Emerging evidence suggests that alterations in stem cell fate significantly contribute to SONFH progression. Cell death pathways such as apoptosis, ferroptosis, necroptosis, and dysregulated autophagy in osteoblasts, adipocytes, and chondrocytes can also exacerbate the development of SONFH. Studies investigating the cell niche alternations in SONFH are shown in Table 2.

**Table 2:** Studies investigating the cell niche alternations and metabolism disorders in SONFH

Reference	Cell niche changes	Animals	Involved cells	Relating pathways	Evaluation methodology	Outcome
Adapala et al.^64^	Macrophage polarization	Yorkshire piglets	Macrophages generated from bone marrow	TLR4 activation	Micro-CT, histology, immunohistochemistry, and qRT-PCR	Necrotic bone induced pro-inflammatory responses in the macrophages through TLR4 activation.
Luvanda et al.^66^	Macrophage polarization	/	THP-1 cells	PKM2 pathway	Immunofluorescence	Dex-mediated phenotypic changes were associated with a metabolic switch in macrophages via PKM2.
Nicolaidou et al.^210^	Macrophage differentiation	/	Monocytes	STAT3 pathway	Histology	Monocytes played a critical role in osteogenic differentiation via OSM production and the induction of STAT3 signaling in MSCs.
Zhang et al.^80^	Inflammatory dysregulation	/	Circulating B cells	/	Flow cytometry	The percentages of CD86^+^ CD19^+^ B cells were positively associated with the degree of femoral head collapse induced by steroid.
Wang et al.^81^	Inflammatory dysregulation	/	Neutrophils, B cells	/	Single-cell RNA sequencing	Inflammatory pathways relating to B cells were clearly altered in SONFH.
Zou et al.^72^	Inflammatory cytokine disorder	/	/	/	Immunochemistry, ELISA	Th17 and IL-17 significantly increased in SONFH synovial fluid which might be responsible for the clinical syndrome.
Geng et al.^74^	Inflammatory cytokine disorder	/	/	IL-9/JAK/STAT pathway	Cytometric bead array (CBA) analysis	IL-9 elevated the presence of inflammation and enzymes related to breaking down cartilage matrix, while also intensifying the activation of JAK-STAT signaling.
Ren et al.^73^	Inflammatory cytokine disorder	/	/	IL-17 and TNF signaling pathways	Gene Ontology (GO) and Kyoto Encyclopedia of Genes and Genomes (KEGG) enrichment analyses	Inflammatory cytokines were crucial treatment targets for SONFH.
Cao et al.^84^	Adipocyte differentiation	/	3T3-L1 cells	/	WB	C/EBP expression might be responsible for adipocyte differentiation.
Kim et al.^85^	Enhanced adipogenesis	/	MSCs	/	Histology, immunofluorescence	sLZIP enhanced formation of the PPARγ2 corepressor complex and thus regulates adipogenesis.
Koromila et al.^86^	Enhanced adipogenesis	/	ST2/Rx2^dox^ cells	/	Immunofluorescence, immunoprecipitation assay, luciferase assay	The inhibitory effect of Runx2 by GCs was mainly caused by the interaction between GR and Runx2.
Song et al.^67^	Unbalanced osteoclastogenesis	Rabbits	/	/	Histology, RT-PCR	Wenyangbushen formula could significantly elevate the expression of VEGF and OPG while inhibiting RANK and RANKL.
Youm et al.^99^	Increased apoptosis	/	/	/	Histology	Apoptosis might play an important role in SONFH.
Kothapalli et al.^100^	Increased apoptosis	Piglets	/	/	Histology, transmission electron microscopy studies	Both apoptosis and oncosis were demonstrated to cause ischemic injury to the femoral head.
Zhu et al.^101^	Increased apoptosis	Rats	MC3T3-E1 cells	/	Histology	GC-induced apoptosis might result in SONFH.
Zheng et al.^104^	Increased apoptosis	/	/	p38 MAPK/NF-κB pathway	Histology, qRT-PCR, and Flow cytometry analysis	Elevation of TNF-α would promote apoptosis and autophagy through the p38 MAPK/NF-κB signaling pathway.
Zhu et al.^105^	Increased apoptosis	/	MC3T3-E1 cells	Chk2/p53 pathway	WB, RT-PCR	Dex stimulated apoptosis in osteoblast via Chk2/p53 pathway.
Zuo et al.^102^	Increased apoptosis	/	HUVECs	p53 pathway	High-throughput RNA sequencing, transwell chamber assay	JMY upregulation induced by GCs triggered apoptosis and decrease of mobility in HUVECs.
Zhang et al.^103^	Increased apoptosis	Rats	BMECs	FAR591/Fos pathway	LncRNA cluster assay, immunofluorescence, histology	FAR591 would target Fos and thus stimulating the apoptosis in SONFH patients.
Chen et al.^106^	Increased ferroptosis	/	/	/	KEGG and GO	27 ferroptosis-related DEGs were identified to be involved in SONFH.
Sun et al.^107^	Increased ferroptosis	/	MC3T3-E1cells, MOLY4 cells	P53/SLC7A11/GPX4 pathway	KEGG and GO, transmission electron microscopy	Dex-induced MC3T3-E1cells ferroptosis through the p53/SLC7A11/GPX4 pathway.
Xu et al.^108^	Increased necroptosis	/	BMECs	RIPK1/RIPK3/MLKL pathway	Network pharmacology analysis, transwell assay, wound-healing assay	Luteolin ameliorated necroptosis in SONFH patients by the RIPK1/RIPK3/MLKL pathway.
Feng et al.^111^	Increased necroptosis	Rats	/	RIPK1/RIPK3/MLKL pathway	Immunohistochemistry, histology, transmission electron microscopy	Necrostatin-1 prevented necroptosis through the RIP1/RIP3/MLKL pathway.
Liang et al.^113^	Decreased autophagy	/	/	/	KEGG and GO	34 potential autophagy-related genes were identified to be involved in SONFH.
Zhu et al.^114^	Decreased autophagy	/	MLO-Y4 cells	/	KEGG and GO	PTH was demonstrated to be protective against Dex-induced apoptosis.
Huang et al.^115^	Decreased autophagy	/	/	/	Microarray, KEGG, and GO	PTHR1 might be involved in the pathology of SONFH via interacting with VDR.
Wang et al.^120^	Adipose metabolism disorder	/	MSCs, adipocytes	/	Histology	The amount of adipocytes among the MSC population of the SONFH group was increased.
Wang et al.^119^	Adipose metabolism disorder	Rabbits	Adipocytes	/	Histology	The increase of the volume of adipocyte might result in the elevation of tissue pressure of femoral head.
Yu et al.^116^	Adipose metabolism disorder	/	/	/	/	Many types of lipid were increased in ONFH patients.
Yan et al.^121^	Adipose metabolism disorder	/	/	/	Ultra-high performance liquid chromatography-tandem mass spectrometry (UHPLC-MS/MS), Bi-clustering heatmap	Different signatures of elevation of lipids existed in different types of ONFH.
Mei et al.^117^	Adipose metabolism disorder	Rabbits	/	/	Ultra-High-Performance Liquid Chromatography, MRI, histology	GC could regulate the metabolic disorder of endogenous lipids in SONFH rabbits.
Xu et al.^125^	ROS accumulation	Rats	MSCs	NOX/ROS/NF-κB pathway	Radiographical (µCT) scanning, histology, immunohistochemistry	Imbalanced apoptosis and differentiation due to GCs played a key role in causing SONFH by activating the NOX/ROS/NF-κB signaling pathway.
Kömürcü et al.^132^	ROS accumulation	Rats	/	/	Hematological examinations, histology	Coenzyme Q10 might be useful as a preventing agent in SONFH by elevating the expression of antioxidants.
Chen et al.^124^	ROS accumulation	Rats	/	/	Micro-CT, Histology, ROS fluorescence	GC treatment enhanced the expression of osteoclast-related substances while downregulating the antioxidants.
Chen et al.^130^	ROS accumulation	Rats	BMSCs	AMPK/PGC-1α/SIRT3 pathway	Immunofluorescence, viral transfection, SOD activity assay	GC treatment could impair BMSCs' osteogenic differentiation was via downregulation of the AMPK/PGC-1α/SIRT3 axis.
Fang et al.^131^	ROS accumulation	Rats	MC3T3-E1 cells, HUVECs	/	Micro-CT, histology	SIRT6 was demonstrated to suppress ferroptosis and restore bone formation and angiogenesis capabilities.
Bai et al.^128^	ROS accumulation	/	MC3T3‑E1 cells	ASK1-p38 pathway	Annexin V-FITC/PI assay, WB	NOX1- and NOX4-derived ROS played a pivotal role in high-dose Dex-induced preosteoblast apoptosis.
Fan et al.^129^	ROS accumulation	Rats	/	/	Dynamic MRI, histology	GC-induced NOXs (including NOX-2) expression might be an important source of oxidative stress.
Fan et al.^133^	Ros accumulation	/	MC3T3-E1 cells	ROS/JNK pathway	Fluorescence microscopy, histology	Hyp decreased the activation of the ROS/JNK pathway in DEX-induced cells, which was then counteracted by NOX4 overexpression.
Yang et al.^134^	ROS accumulation	/	BMSCs	Keap1/Nrf2 pathway	Immunofluorescence	MAGL inhibition regulated oxidative stress in BMSCs via the Keap1/Nrf2 pathway, thus mitigating SONFH.
Zhang et al.^144^	Collagen synthesis	Yorkshire piglets	COS7 cells	SOX9 pathway	Histology, coimmunoprecipitation assay	HIF-1α activated Sox9 expression and elevated Sox9-mediated transcriptional activity.
Lin et al.^135^	NO synthesis	/	BMSCs	Ankyrin-Akt-eNOS pathway	Immunofluorescence analysis, histology	The decreased ankyrin expression in ONFH patients could result in abnormal eNOS signaling, reduction in cell proliferation, and poor osteogenic differentiation potential in BMSCs.
Calder et al.^136^	NO synthesis	/	/	/	Histology	Aberrant NO production in patients with osteonecrosis led to defective osteoblastogenesis.

### Endothelial cell disorder

**Fig. 3:**
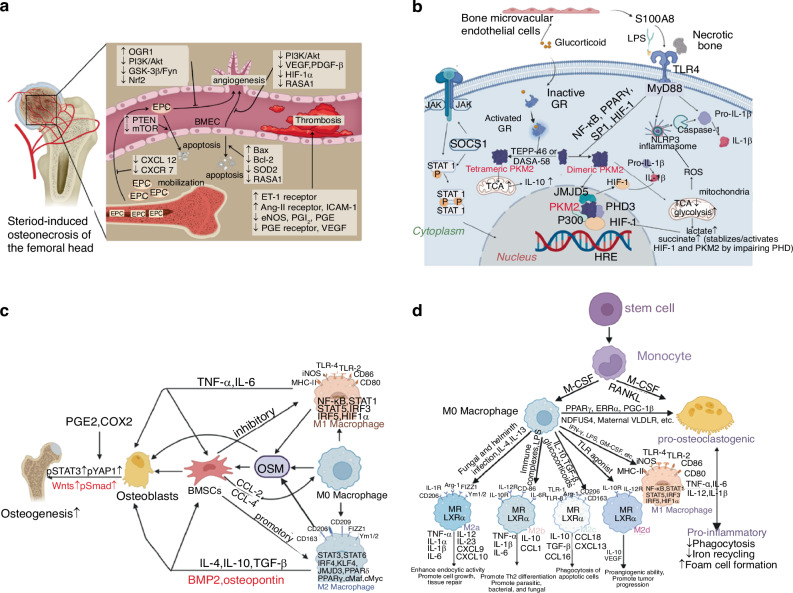
CCellular mechanisms underlying SONFH. a Pathogenesis in SONFH correlating with bone endothelial cells. **a** Pathogenesis in SONFH correlating with bone endothelial cells. SONFH was affected by GCs' altered mobilization, angiogenesis, apoptosis, and thrombosis of bone endothelial cells through several signaling pathways or cytokines like PI3K/Akt, GSK-3β/Fyn, Bcl-2, and Bax. Reproduced with permission. Copyright 2021, Huang et al.^9^ **b** Activation of M1 polarized macrophage in the development of GIONFH, which mainly included the IFN-γ and TLR4 pathways. Reproduced with permission. Copyright 2023, Zhang et al.^63^ **c** Association between osteogenesis and macrophage polarization in the development of GIONFH, showing that the M0, M1, and M2 phenotypes were all beneficial for osteogenesis by stimulating the OSM signaling pathway. Reproduced with permission. Copyright 2023, Zhang et al.^63^ **d** Molecular determinants between the differentiation and polarization of macrophages, and the differentiation and maturation of osteoclasts, which were the result of competing differentiations of myeloid progenitors. Reproduced with permission. Copyright 2023, Zhang et al.^63^

The disorder and destruction of bone endothelial cells have been extensively implicated in SONFH. Huang et al.^9^ reported that GC-induced damage to bone microvascular endothelial cells (BMECs) and endothelial progenitor cells (EPCs) strongly correlates with the SONFH progression. GCs contribute to this damage partially by activating the mitochondrial apoptotic pathway in BMECs by increasing the expression of pro-apoptotic B-cell lymphoma-2(Bcl-2)-associated X (Bax) while decreasing the expression of anti-apoptotic Bcl-2 and Ras p21 protein activator 1 (RASA1).^51,52^ Nevertheless, BMECs are categorized into two types: H-type and L-type, of which H-type exhibits greater proliferative capacity and is closely associated with osteoprogenitor cells.^53^ However, the role of these BMEC subtypes in SONFH pathogenesis remains unclear. On the other hand, endothelial progenitor cells (EPCs) can also be classified into two groups: early EPCs (eEPCs) and late EPCs (lEPCs).^54^ eEPCs exhibit a stronger secretory ability, producing growth factors such as vascular endothelial growth factor (VEGF),^55^ while lEPCs display greater proliferative capacity.^56^ Research shows that GC treatment significantly impairs eEPCs' mobility by downregulating chemokine receptor type 4 (CXCR4) expression.^57,58^ Moreover, ovarian cancer G-protein coupled receptor 1 (OGR1) negatively affects EPCs mobility, compounding the detrimental impact of GCs treatment on EPCs function (Fig. 3a).^9^

### Osteoimmunology

The term “osteoimmunology” describes the bidirectional interactions between osteoblasts, osteoclasts, and immune cells. Osteoblasts and osteoclasts not only respond to immune signals such as interleukins (ILs) and interferon-γ (IFN-γ) but also actively regulate immune cell differentiation within the bone marrow niche through secreting chemokines, IL-7, and other factors.^59^ This mutual and dynamic relationship between immune cells and bone-related cells underpins the immunopathology of SONFH.

#### Macrophage polarization and differentiation

Macrophages are the first immune cells to respond in inflammatory areas and play a key role in SONFH pathogenesis. During the initial response, M1 macrophages predominate, releasing cytokines such as tumor necrosis factor-α (TNF-α), IL-1β, and IL-6, which promote osteocyte apoptosis and inhibit tissue repair.^60^ Prolonged M1 activation leads to chronic inflammation and progressive femoral head damage. Failure to transition to the M2 macrophage phenotype, which is responsible for tissue repair and angiogenesis, further exacerbates disease progression.^61^ Studies have demonstrated that therapeutic repolarization from M1 to M2 macrophages, for example via Astragaloside IV treatment, can mitigate SONFH progression.^62^ The differentiation bias between M1 and M2 macrophages progresses in distinct phases during SONFH. Necrotic tissue releases damage-associated molecular patterns (DAMPs) that bind to Toll-like receptor 4 (TLR4), leading to the production of Th1 cytokines such as IFN-γ and TNF-α, as well as hypoxia-inducible factor-1α (HIF-1α), which together promote fibrovascular tissue formation and the accumulation of M1 macrophages (Fig. 3b).^61,63,64^ Although GCs are known to suppress immune reactions,^65^ they do not directly induce this imbalance. Instead, GCs can enhance M2 macrophage differentiation during later stages, aiding bone tissue repair by promoting the secretion of BMP2 (Fig. 3c).^63,66^ However, during the active phase of SONFH, GCs can damage BMECs, leading to increased stabilization of HIF-1α and IL-1β, which skews macrophage polarization toward the M1 subtype, further exacerbating local inflammation and tissue damage.^63,66^

Macrophages give rise to osteoclasts, creating a competitive differentiation process between M1/M2 macrophages and osteoclasts. Elevated osteoclast activity is generally observed at the boundary of necrotic regions in the femoral head, potentially leading to in situ fractures.^31^ In necrotic femoral head tissue, osteoprotegerin (OPG) expression is downregulated, while the expression of receptor activator of nuclear factor-κB (RANK) and its ligand RANKL is upregulated, promoting osteoclastogenesis and disrupting bone remodeling.^67^ In patients with SONFH, excessive GCs have been shown to directly enhance the differentiation of macrophages into osteoclasts via the RANKL/nuclear factor kappa-light-chain-enhancer of activated B cells (NF-κB) pathway.^68,69^ Further research has revealed that GCs, together with the RANKL pathway, increase the production of reactive oxygen species (ROS), thereby further promoting osteoclastogenesis.^70^ Moreover, since GCs have been reported to promote the differentiation of M1 macrophage, osteoclastogenesis is indirectly promoted by GCs through M1-related peroxisome proliferator-activated receptor γ (PPARγ), TNF-α, IL-6, IL-1, IL-12, and chemokines such as CXCL2, CXCL8, and CXCL10,^71^ while factors produced by M2 macrophages, such as NDUFS4, can inhibit osteoclast differentiation (Fig. 3d).^63^

#### Lymphocytes and neutrophils

**Fig. 4:**
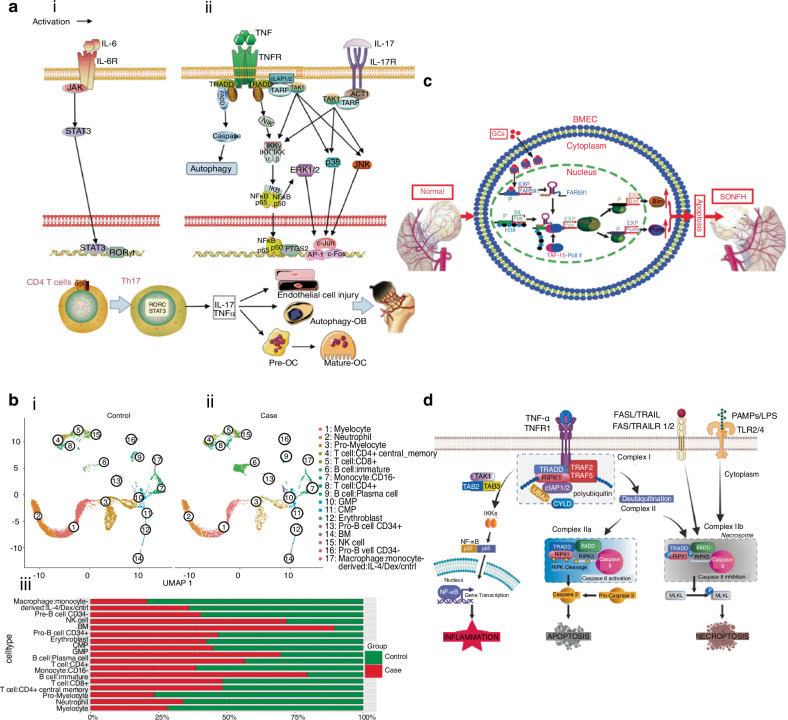
Cellular process underlying SONFH. **a** Action mechanism of IL-17 and TNF signaling pathways. CD4 T cells on the left (i) activated the expression of RORγt through the IL-6/JAK/STAT3 signal and differentiated into Th17 cells. TNF on the right (ii) could bind to TNFR and interact with the death domain (TRADD) to recruit molecules like TRAF, RIP1, FADD, and TAK1and thus regulate the downstream pathways. Reproduced with permission. Copyright 2022, Ren et al.^73^ **b** Profile of cell types and corresponding cell number and proportion. The distribution of the same 17 cell types in both the case and control groups (i, ii). Comparison of the proportion of the same cell type in the case and control group (iii). Reproduced with permission. Copyright 2023, Wang et al.^81^ **c** Schematic diagram of the mechanism of FAR591-mediated SONFH. Reproduced with permission. Copyright 2023, Zhang et al.^103^ **d** A diagram shows TNF-α/TNFR1 signaling sets off necroptosis while also instigating apoptosis and inflammation. A deeper dive into the molecular intricacies of TNF-α/TNFR1 signaling revealed its complexity. Furthermore, various other signaling pathways, such as FASL/FAS, TRAIL/TRAIL, PAMPS, or LPS/TLR2/4 were capable of initiating necroptosis. Reproduced with permission. Copyright 2021, Elsevier.^110^

Lymphocytes also play significant roles in the pathology of SONFH. T cells, particularly Th17, Treg, and Th9 subsets, are increasingly recognized as pivotal regulators of inflammation in SONFH. Elevated levels of T helper 17 (Th17) cells in both synovial tissue and peripheral blood of SONFH patients further highlight the link between T cells and disease progression.^72^ Ren et al.^73^ demonstrated that the activation of IL-17 and TNF signaling pathways regulated by Th17 cells is critical in endothelial injury, osteoblast autophagy, and osteoclast proliferation, which collectively drive SONFH progression (Fig. 4a). Th9 and Th17 cells secrete IL-9, which upregulates other inflammation-related cytokines as well as enzymes that degrade cartilage matrix, and has also been found elevated in SONFH.^74,75^ Additionally, inflammatory cytokines like IL-23 and IL-33, produced by Th cells, have been proposed as predictive indicators for SONFH risk.^72,76,77^ Conversely, reduction of regulatory T (Treg) cells may be linked to SONFH progression,^78^ for Treg cells exhibit anti-inflammatory properties via secreting IL-10 and TGF-β, suppressing osteoclast activity by releasing anti-inflammatory cytokines, and binding to osteoclast precursors.^59^ B cells are also implicated in SONFH pathogenesis by inducing humoral immune responses.^79,80^ Single-cell RNA sequencing revealed substantial dysregulation in B cells within SONFH patients (Fig. 4b).^81^ Activated B cells have been reported to contribute to ONFH through pro-inflammatory cytokine secretion (e.g., IFN-γ, IL-17A) and by promoting osteoclastogenesis.^61^ Neutrophils infiltrate necrotic femoral head tissue in early ONFH and form neutrophil extracellular traps (NETs), which impair local blood flow and contribute to thrombus formation.^82^

In summary, SONFH is increasingly recognized as an osteoimmune disorder. GCs disrupt immune homeostasis by promoting M1 macrophage polarization, suppressing regulatory T cell function, and enhancing the pro-inflammatory activity of Th17 and B cells.^60,61^ These changes contribute to excessive osteoclastogenesis, vascular damage, and bone cell apoptosis through cytokine-driven pathways such as RANKL/OPG, NF-κB, IL-6/JAK-STAT3, and TLR4 signaling.^60,61^ Importantly, this immune imbalance evolves dynamically throughout disease stages---from acute sterile inflammation and microvascular injury to chronic necrosis and bone collapse. Crosstalk between immune cells and bone-resident cells amplifies tissue damage and hinders regeneration, establishing a self-perpetuating inflammatory-osteolytic loop.^60,61,63,66,73–75^ Targeting the immune microenvironment by modulating macrophage polarization or restoring the Th17/Treg balance holds promise as a novel therapeutic approach, particularly in the early stages of SONFH.

### Stem cell fate alteration

Bone marrow mesenchymal stromal cells (BMSCs) are multipotent cells capable of differentiating into various cell types, including bone, fat, and cartilage.^83^ The process of adipogenesis is a key competitor against osteoblast generation. GCs treatment directly upregulates adipogenic transcription factors such as PPARγ and CCAAT/enhancer-binding protein α (C/EBPα).^84,85^ Additionally, GCs also directly inhibit osteoblastogenesis by downregulating Runt-related transcription factor 2 (Runx2), a key osteogenic inducer, thereby skewing BMSCs toward adipocyte differentiation.^86^ Of note, low concentrations of GCs (10^−^^8^–10^−9 ^mol/L) can promote BMSCs proliferation and osteoblastogenesis, but pharmacological doses (10^−^^7^–10^−6 ^mol/L) suppress osteogenesis and favor adipogenesis by inhibiting the Wnt/β-catenin signaling pathway.^87–92^ The Notch signaling pathway, essential for determining cell fate, can also be activated by GC stress.^93,94^ While normal Notch1 expression enhances osteogenesis, excessive Notch1 expression induced by GCs paradoxically promotes osteonecrosis.^95,96^

### Programmed cell death

Disordered programmed cell death across multiple cell types was notably observed in SONFH, of which apoptosis, ferroptosis, necroptosis, and autophagy are most extensively involved.

Apoptosis plays a significant role in SONFH, with studies demonstrating that femoral head collapse results from extensive apoptosis of bone and endothelial cells.^97–100^ GCs induce apoptosis predominantly through the mitochondria-mediated pathway.^101^ GCs can also enhance the expression of TNF-α and p53 signaling pathway, which both have been implicated in the induction of osteoblast and BMECs apoptosis (Fig. 4c).^102–105^ Moreover, inhibition of the PI3K/Akt pathway by GCs has been shown to enhance apoptosis and contribute to SONFH progression.^9^

Ferroptosis, characterized by ROS accumulation and iron-dependent lipid peroxidation, is another form of regulated cell death implicated in SONFH. Transcriptional analyses have identified HIF-1, TNF, and Forkhead box O1 (FOXO1) signaling as pathways associated with ferroptosis contributing to SONFH.^106^ Further studies confirmed that dexamethasone (DEX) induces ferroptosis in osteoblasts by upregulating p53 and downregulating SLC7A11 and GPX4 expression.^107^

Necroptosis, a form of regulated cell death characterized by cell rupture and inflammation, also contributes to SONFH. GC-induced TNF-α amplifies necroptosis by triggering both apoptotic and inflammatory pathways (Fig. 4d).^108–111^ Notably, the use of necroptosis inhibitors has been reported to mitigate the progression of SONFH.^111,112^

Autophagy is a protective process that involves the degradation and recycling of cellular components to maintain homeostasis. Bioinformatics analyses have linked autophagy-related genes to the pathogenesis of SONFH.^113^ While low doses of DEX (10^−6 ^mol/L) enhance autophagy, higher doses (10^−5 ^mol/L) inhibit autophagy while promoting apoptosis.^113^ Notably, PTH has been shown to alleviate DEX-induced apoptosis by enhancing autophagy, suggesting a potential therapeutic strategy to reduce the risk of SONFH.^114,115^

## Metabolism disorder

Physiological levels of GCs play an essential role in regulating metabolism by promoting protein and lipid degradation, maintaining normal blood glucose levels, and inhibiting fat synthesis. However, prolonged high-dose GC treatment disrupts these normal functions, leading to various metabolic imbalances that contribute to SONFH development. Studies investigating the metabolic disorder in SONFH are shown in Table 2.

### Lipid metabolic disorder

**Fig. 5:**
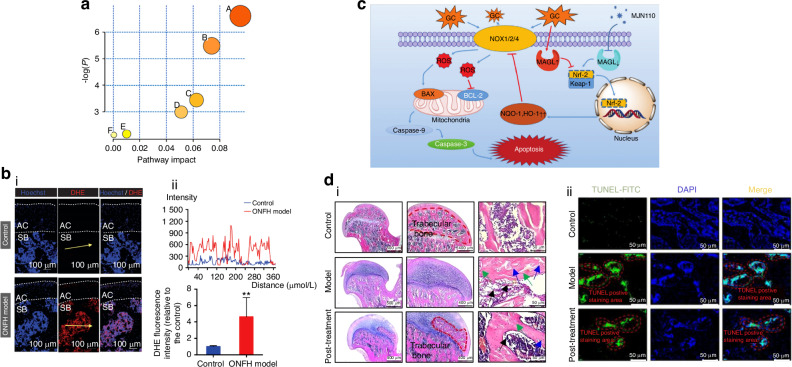
Metabolism disorders associated with SONFH. **a** Metabolic pathway analysis was conducted on serum samples from the GIONFH rabbit model, focusing on (A) glycerophospholipid metabolism, (B) sphingolipid metabolism, (C) linoleic acid metabolism, (D) alpha-Linolenic acid metabolism, (E) pyrimidine metabolism, and (F) arachidonic acid metabolism. Reproduced with permission. Copyright 2022, Mei et al.^117^ **b** Analysis of reactive oxygen species (ROS) level and antioxidant enzymes in the rat femoral head. (i) Cryosections of femoral heads were examined to assess the levels of ROS using dihydroethidium (DHE) probing in both the control and model groups. Regions of interest included the articular cartilage (outlined by white dashed lines; AC) and the subchondral bone (SB). (ii) DHE fluorescence intensity profile through the subchondral area of the femoral head (indicated by the yellow arrow in (i). The horizontal axis of the DHE intensity profile indicated the distance from the initial point marked by the yellow arrow. And quantitative analysis of DHE fluorescence intensity relative to the control group (*n* = 5 in each group). Reproduced with permission. Copyright 2020, Chen et al.^124^ **c** A schematic depiction of how MAGL inhibition provides protection against oxidative stress damage induced by GC is presented. MAGL inhibition mitigates the upregulation of GC-induced NADPH oxidative isozymes by activating the Keap1/Nrf2 pathway. Reproduced with permission. Copyright 2021, Yang et al.^134^ **d** Pre-treatment with monoacylglycerol lipase (MAGL) inhibitor alleviates SONFH. (i) Hematoxylin and eosin (H&E) staining. (ii) TUNEL staining of bone tissues. Reproduced with permission. Copyright 2021, Yang et al.^134^

Lipid metabolism influenced by GCs plays a critical role in the development of SONFH.^97^ Patients with SONFH exhibit elevated lipid levels, including total cholesterol (TC), triglycerides (TG), low-density lipoprotein (LDL), and an increased LDL/high-density lipoprotein (HDL) ratio. Conversely, levels of HDL, which are responsible for cholesterol removal, are significantly decreased.^116^ In addition, impaired phospholipid metabolism and lipid oxidation have been identified in SONFH (Fig. 5a).^117,118^ In terms of pathological mechanisms, GCs promote the adipogenic differentiation of BMSCs by upregulating PPARγ expression, leading to the formation of fat emboli that obstruct the microcirculation of the femoral head. Furthermore, the increased diameter of marrow fat cells elevates intraosseous pressure within the confined space of the femoral head, further reducing perfusion and contributing to SONFH development.^119,120^ Plasma lipidomic analysis further shows that palmitate (Palm), which is specifically elevated by GCs, may play a pivotal role in lipid toxicity, leading to BMSCs impairment.^117,121,122^

### Oxidative stress disorder

Oxidative stress plays a pivotal role in the progression of SONFH, with ROS serving as direct instigators of cellular damage.^123^ As key inhibitors of the intracellular antioxidant system, GCs have been shown to activate lipid peroxidation, leading to excessive ROS production and accumulation in BMSCs and osteoblasts, thereby promoting SONFH development (Fig. 5b).^124,125^ Elevated ROS levels, along with associated endoplasmic reticulum stress and apoptosis, have been observed in patients with SONFH.^125–129^ Notably, restoring antioxidant function has been shown to enhance osteoblast survival and differentiation while mitigating the progression of SONFH (Fig. 5c, d).^130–134^

### Nitric oxide (NO) disorder

Nitric oxide (NO) is a critical metabolite that plays a key role in vasodilation and blood pressure regulation. A decrease in NO concentration or sensitivity contributes to vascular occlusion in SONFH. GCs have been shown to reduce the synthesis and sensitivity of endothelial nitric oxide (eNO), primarily through the inhibition of endothelial nitric oxide synthase (eNOS).^9^ Additionally, GCs reduce cytoskeletal proteins such as ankyrin, which further inhibits the eNOS signaling pathway and impairs NO production.^135^ By contrast, elevated expression of inducible nitric oxide synthase (iNOS) has been observed in patients with SONFH, which is thought to prominently promote inflammatory and cell apoptosis.^136^

## Dysregulation in molecular factors and signal pathway cascades

Alterations in molecular factors represent the intrinsic mechanisms driving SONFH pathogenesis. These molecular elements form a complex and interconnected network that disrupts signaling pathway cascades, leading to key pathological processes including impaired vascular repair, heightened inflammation, and an imbalance between osteogenesis and adipogenesis. Studies investigating the pivotal bioactive molecules and the related signal pathways in SONFH are presented in Tables 3 and 4, respectively.

**Table 3:** Studies investigating the pivotal bioactive molecules in SONFH

Reference	Animals	Involved molecules	Involved cells	Relating pathways	Evaluation methodology	Outcome
Pan et al.^140^	Rabbits	VEGF	/	TGF-β1/BMP2 pathway	Histology, immunofluorescence	The decreased level of VEGF in the femoral head significantly decreased the expression of TGF-β1 and BMP2.
Song et al.^141^	Rabbits	VEGF	BMSCs	/	MRI, histology	Co-culture of healthy BMSCs with ONFH BMSCs could significantly increase the expression of VEGF.
Zhang et al.^142^	Rats	VEGF	EAhy926 cells, MG63 cells	/	Histology, 3-D microangiography.	Vitamin K2 was capable of promoting angiogenesis by elevating VEGF.
Zhang et al.^143^	Rats	VEGF	BMSCs	/	Histology, micro-CT, immunohistochemistry	VK2 showed a significant antagonist effect for GC on osteogenic progenitors.
Gao et al.^138^	Rats	VEGFR	/	/	Histology, micro-CT	VEGF receptor 2 antibody could induce ONFH.
de Campos Pessoa et al.^139^	Rats	VEGF	/	NO pathway	Immunohistochemistry, histology	Phosphodiesterase-5 inhibition enabled the protective effect of VEGF at the early stage of ONFH.
Arnett et al.^152^	Rats	HIF-1α	BMSCs	/	Histology	Hypoxia-induced HIF-1α might be involved in bone resorption through regulating the genesis of osteoclasts.
Zhu et al.^153^	/	HIF-1α	MLO-Y4 cells	JAK2/STAT3 pathway	Immunofluorescence, TRAP staining	HIF-1α promoted the expression of RANKL via activating the JAK2/STAT3 pathway.
Song et al.^154^	/	HIF-1α	MLO-Y4 cells	JNK/caspase-3 pathway	Immunofluorescence, histology	HIF-1α served as a pro-apoptotic factor by activating the JNK/caspase-3 signaling pathway.
Wang et al.^147^	Mice	HIF-1α	Type H endothelium	Notch pathway	Immunofluorescence, micro-CT	HIF-1α played a pivotal role in coupling the proliferation of H-type endothelium cells and osteogenesis.
Jiang et al.^148^	Rats	HIF-1α	BMSCs, HUVECs	p38/MAPK pathway	Scratch wound assay, tube formation assay	MMP-2 inhibitor 1 (MMP-2-I1) would promote osteogenesis of BMSCs via the p38/MAPK pathway and angiogenesis of HUVECs through the HIF-1α signal.
Yamaguchi et al.^151^	Piglet	HIF-1α	/	/	Immunofluorescence, histology	Ischemia induced the elevation of IL-6 and inflammation through the HIF-1α-dependent way.
Xu et al.^150^	Rats	HIF-1α	MLO-Y4 cells	Mitophagy pathway	Immunofluorescence, WB, flow cytometry	HIF-1α regulated its downstream marker BNIP3 and enhanced mitophagy inhibited by hypoxia.
Hawker et al.^157^	/	Intravascular sickling	/	/	Histology	Intravascular sickling and the increased viscosity were both revealed to be related to the development of SONH.
Akinyoola et al.^158^	/	Intravascular sickling	/	/	Clinical data	Not all patients with sickle cell disease had impaired fibrinolytic activity.

**Table 4:** Studies investigating the related signal pathways in SONFH

Reference	Animals	Involved cells	Relating pathways	Evaluation methodology	Outcome
Fang et al.^179^	/	BMSCs	BMP/Runx2 pathway	ELISA, immunohistochemistry	TNFα promoted cell proliferation and angiogenesis, whereas it inhibited osteogenesis by binding to H3K27me3 of the Runx2 promoter.
Kuribayashi et al.^182^	/	/	BMP/Runx2 pathway	Case-control study	ABCB1 might interact with the GC receptor and thus stimulate SONFH.
Han et al.^180^	Rats	BMSCs	BMP/Runx2 pathway	Flow cytometry, micro-CT	Overexpression of P-gp inhibited BMSC adipogenesis and promoted osteogenesis mainly through the BMP2/Runx2 pathway.
Han et al.^181^	Rats	BMSCs	BMP/Runx2 pathway	Histology, immunofluorescence	Enhanced expression of P-gp alleviated the process of SONFH through the BMP2/Runx2 pathway.
Ye et al.^184^	Rats	/	VEGF/Runx2/BMP2 pathway	Histology, ELISA	Ginsenoside Rb1 attenuated SONFH through the VEGF/RUNX2/BMP‑2 pathway.
Xu et al.^183^	Mice	/	BMP/Runx2 pathway	Immunohistochemistry, micro-CT	EPO prevented bone loss in ONFH in mice via enhancing Runx2‑mediated osteogenesis,
Duan et al.^186^	Rats	BMSCs	PPARγ pathway	Histology, micro-CT	The elevated C/EBPα upregulated the level of histone H3K27 acetylation in the PPARγ promoter region.
Jiang et al.^187^	Rats	BMSCs	PPARγ pathway, Wnt pathway	Immunohistochemistry, micro-CT, RT-PCR	Pravastatin could suppress the PPARγ pathway and activate the Wnt pathway.
Liu et al.^188^	Mice	BMSCs	NIK/NF-κB pathway	Histology, WB	IKKe demonstrated a protective effect of SONFH via the NIK/NF-κB pathway.
Shan et al.^189^	Rats	BMSCs, HUVECs	Akt-related pathway	KEGG and GO, histology, immunofluorescence	AS-IV prevented SONFH by promoting osteogenesis and angiogenesis through the Akt/Runx2 and Akt/HIF-1α/VEGF pathways, and suppressing apoptosis and ROS via the Akt/Bad/Bcl-2 and Akt/Nrf2/HO-1 pathways.
Shen et al.^201^	Rats	BMSCs	PI3K/Akt-Bax/Bcl-2/caspase-3 pathway	Histology, immunofluorescence	Vinp activated the PI3K/Akt pathway in osteoblasts and thus inhibited apoptosis.
Wang et al.^195^	/	RAW 264.7 cells	PI3K/Akt pathway	KEGG and GO, histology, immunofluorescence	BTP administration could induce PI3K/Akt-mediated apoptosis in osteoclasts to treat SONFH.
Lu et al.^190^	Rats	MC3T3-E1 cells	PI3K/Akt pathway	Flow cytometry, KEGG, and GO, microarray analysis	FGF-2 was revealed to suppress Dex-induced apoptosis through promoting the PI3K/Akt pathway.
Larsen et al.^191^	Rats	/	/	Scanning electron microscopy	FGF-2 was demonstrated to enhance surgical angiogenesis in vascularized bone.
Jiang et al.^205^	Mice	Mouse embryonic fibroblast cells (MEFs)	PI3K/Akt/COX-2 pathway	Micro-CT, histology, immunofluorescence	IGF-1 might reverse the osteogenic inhibitory effect of Dex via the PI3K/AKT pathway through activating COX-2.
Zou et al.^192^	Mice	MC3T3-E1 cells	PI3K/Akt pathway	RT-PCR, WB	GCs promoted the level of p85α and inhibited the PI3K/Akt pathway to induce apoptosis.
Lv et al.^200^	Rats	/	PI3K/Akt pathway	Histology, WB	TFRD protected osteoblasts from Dex‑induced damage through the PI3K/AKT pathway.
Jang et al.^197^	Rats	VECs	PI3K/Akt/mTOR pathway	Immunofluorescence, electron microscope	GCs inhibited the protective effect of autophagy on SONFH by increasing the expression of the PI3K/Akt/mTOR pathway.
Yu et al.^204^	Rats	BMSCs	PI3K/Akt pathway	Immunofluorescence	CPA could ameliorate the downregulation of osteogenesis by alcohol, partly via the PI3K/AKT pathway
Wang et al.^196^	Rats	MLO-Y4 cells	PI3K/Akt/mTOR pathway	WB, Bioinformatic, immunofluorescence	Pinocembrin alleviated SONFH by activating autophagy by suppressing the PI3K/Akt/mTOR pathway.
Zhu et al.^198^	/	MSCs	CD41-integrin β3-FAK-Akt-Runx2 pathway	Histology, micro-CT, KEGG, and GO	The exosomes from the necrotic bone lacked CD41, which would then alleviate the osteogenesis and MSCs migration through downregulating the CD41-integrin β3-FAK-Akt-Runx2 pathway.
Xu et al.^202^	/	BMSCs	Akt/Bad/Bcl‑2 pathway	Microarray analysis, histology, immunofluorescence	LINC00473 was capable of rescuing BMSCs from Dex‑induced apoptosis through the PEBP1‑mediated activation of the Akt/Bad/Bcl‑2 pathway.
Tao et al.^203^	Rats	MC3T3-E1 cells, HMEC-1 cells	Akt/Bad/Bcl‑2 pathway	Tube formation assay, micro-CT, histology	PRP-Exos rescued the GC-induced apoptosis through the Akt/Bad/Bcl-2 pathway.
Xu et al.^199^	/	BMSCs	miR-23a-3p/PEBP1/Akt/Bad/Bcl-2 pathway, miR-23a-3p/LRP5/Wnt/β-catenin pathway	Microarray analysis, histology, immunofluorescence	LINC00473 could promote osteogenesis and suppress the adipogenesis of BMSCs through the activation of the miR-23a-3p/LRP5/Wnt/β-catenin pathway, while alleviating Dex-induced apoptosis by activating the miR-23a-3p/PEBP1/Akt/Bad/Bcl-2 pathway.
Chen et al.^193^	/	BMSCs	GDF15/Akt/mTOR pathway	Immunofluorescence	DEX inhibited the proliferation and induced apoptosis of BMSCs by suppressing the GDF15/Akt/mTOR pathway.
Li et al.^194^	Rats	BMSCs	GDF15 pathway	Histology, immunofluorescence, micro-CT	Melatonin ameliorated ferroptosis by regulating the GDF15 pathway.
Li et al.^160^	Mice	/	TGF-β/Smad pathway	ELISA, immunohistochemistry	These results suggested that macrophage-derived TGF-β1 recruits MSCs to differentiate into myofibroblasts.
Wang et al.^161^	Mice	Mesenchymal stromal/progenitor cells (MSPCs)	TGF-β/Smad pathway	Micro-CT, immunohistochemistry	Active TGF-β promoted the ectopic MSPCs recruitment and bone formation.
Zhen et al.^170^	Mice	BMSCs	TGF-β/Smad pathway	Micro-CT, micro–MRI, immunohistochemistry	Active TGF–β1 in the subchondral bone initiated the pathological changes of osteoarthritis.
Lu et al.^165^	/	BMSCs	TGF-β/Smad pathway	RT-PCR, ChIP assay	Smad4 activated the transcription of GCN5 to inhibit apoptosis and promote osteogenesis.
Okada et al.^167^	/	/	TGF-β/Smad pathway	RT-PCR	Puerarin promoted the osteogenesis process by stimulating the expression of Smad2/3.
Elsafadi et al.^166^	/	MSC-TERT cells	TGF-β/Smad pathway	Histology, immunofluorescence	There was a reciprocal relationship between the TGF-β/Smad2/3 pathway and the BMP pathway, which would both facilitate bone formation.
Cui et al.^169^	Mice	Bone marrow macrophage	MiR-21-5p/Smad7 Pathway	Histology, immunofluorescence	MiR-21-5p directly targeted Smad7, which eventually led to the activation of fibrogenesis in tendon cells.
Huang et al.^175^	/	MSCs	Wnt/β-catenin pathway	RT-PCR, immunofluorescence	GSK-3β was able to attenuate the osteogenesis process of MSCs.
Nie et al.^176^	Rats	/	Wnt/β-catenin pathway	WB, micro-CT	GC-induced SONFH in rats through activating the GSK-3β-mediated osteoblast apoptosis.
Cheng et al.^228^	/	BMSCs	Wnt/β-catenin pathway	Histology, immunofluorescence	m6A level of PTPN6 was decreased in SONFH patients, leading to the release of GSK-3β and its inhibitory effect of the Wnt/β-catenin pathway.
Sun et al.^177^	Rats	BMSCs, HUVECs	Wnt/β-catenin pathway	Histology, immunofluorescence, micro-CT	Activation of CB2 promoted osteogenesis through the activation of the Wnt/β-catenin pathway.
Chen et al.^174^	/	BMSCs	BMP2-Wnt/β-catenin pathway	qPCR, WB	Polydatin promoted osteogenesis through the BMP2-Wnt/β-catenin signaling pathway.
Nie et al.^206^	/	/	/	qRT-PCR, gene chip assay	GSK-3β shRNA might affect the expression of various apoptosis-related genes.
Ding et al.^208^	/	EPCs	Akt/GSK-3β/Fyn pathway	Immunofluorescence, tube formation assay	The angiogenesis function of EPCs was affected by the downregulation of CXCR7 and its downstream Akt/GSK-3β/Fyn pathway.
Xing et al.^211^	Rats	/	RELA/Akt1 pathway	KEGG and GO, RT-PCR	Duhuo Jisheng Decoction inhibited the activity of osteoclasts in SONFH through downregulating the RELA/Akt1 pathway.
Geng et al.^74^	/	/	JAK/STAT pathway	MRI, immunohistochemistry	IL-9 not only induced inflammation but also enhanced the expression of the JAK/STAT pathway, which would worsen the condition of SONFH.
Chen et al.^212^	/	/	JAK/STAT pathway	Immunohistochemistry, ELISA	The IL-21-induced degradation of cartilage was also regulated by the activation of the JAK/STAT pathway.

### Molecular factors

#### VEGF

**Fig. 6:**
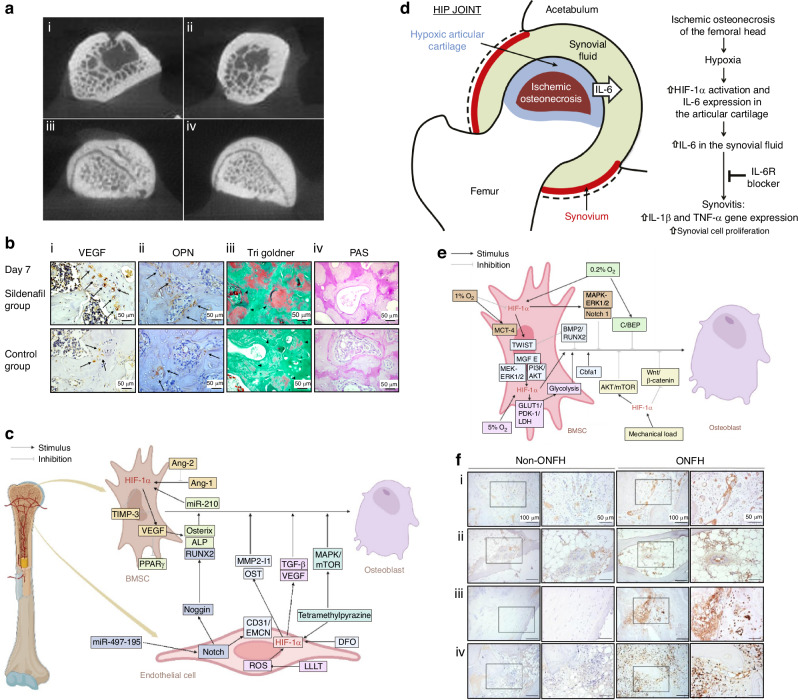
Molecular factors dysregulation in SONFH. **a** Micro-CT examination revealed the contour of the femoral head. In group i (gradually treated with 2 mL of 50 mg/mL VEGFR2 antibody) and group ii (gradually treated with 2 mL of 25 mg/mL VEGFR2 antibody), findings included subchondral cysts, sparse trabecular changes, and deformation of the femoral head. Additionally, group iii (gradually treated with 2 mL of 12.5 mg/mL VEGFR2 antibody) exhibited slight trabecular changes. Conversely, group iv (saline group) displayed tightly packed trabeculae and an intact femoral head. Reproduced with permission. Copyright 2013, Elsevier.^138^ **b** Representative image of the proximal epiphysis of the femur on the 7th day of the experiment. At ×40 magnification, (i) greater VEGF immunostaining was observed in the sildenafil group (black arrows). A similar result was shown for OPN, especially in the regions around the trabeculae (ii). Goldner’s trichrome staining (iii) showed the greater formation of osteoid tissue (the corresponding area is highlighted in red and indicated by black arrowheads, indicative of newly formed and noncalcified bone tissue). Immunohistochemistry (iv) with PAS staining is suggestive of higher production of carbohydrates and suggests higher metabolic activity in the treated group. Reproduced with permission. Copyright 2020, Orthopaedic Research Society.^139^ **c** A figure showing that HIF-1α could couple angiogenesis and osteogenesis. Reproduced with permission. Copyright 2022, Chen et al.^145^ **d** A suggested mechanism of elevated IL-6 levels in the synovial fluid and initiation of synovitis in the hip joint due to HIF-1α following the induction of SONFH. Reproduced with permission. Copyright 2016, The Journal of Bone and Joint Surgery.^151^ **e** A diagram showing hypoxia affects the differentiation of BMSCs into osteoblasts by regulating HIF-1α expression. Reproduced with permission. Copyright 2022, Chen et al.^145^ **f** Immunohistochemical examination of S100A9 in femoral head tissue revealed robust staining in various components. Injured vessels (i), marrow adipose (ii), fibrotic tissue (iii), and osteocytes in cortical bone, as well as inflammatory cells (iv), exhibited intense S100A9 immunostaining. Moreover, there were notable elevations in S100A9-immunostained vessels, adipocytes, fibroblasts, and osteocytes. Reproduced with permission. Copyright 2019, Wu et al.^28^

VEGF plays a critical role in angiogenesis by stimulating endothelial cell growth, proliferation, and migration to sites requiring new blood vessels.^137^ VEGF also increases vascular permeability, facilitating the movement of small molecules and cells from the bloodstream into surrounding tissues, a process known as vascular leakage or vascular permeability. By injecting VEGF receptor 2 antibody, Gao et al.^138^ effectively developed a rat ONFH model (Fig. 6a). By contrast, phosphodiesterase-5 (PDE5) inhibitors can facilitate VEGF-mediated neovascularization and tissue regeneration in damaged bone (Fig. 6b).^139^ This highlights the irreplaceable role of VEGF in maintaining the blood supply of the femoral head. In addition, lower VEGF levels have been detected in necrotic femoral head,^9,67,98,140^ whereas increased VEGF expression has been observed in BMSCs ameliorated SONFH.^141^ Drugs that can elevate VEGF expression, such as vitamin K2 (VK2) and SIRT6, which have been shown to enhance tube formation in endothelial cell lines, thereby mitigating SONFH.^131,142,143^ Furthermore, two low-inducing VEGF haplotypes (C-G-G-C and A-G-G-C) were identified as an important risk factor for SONFH.^7^

#### HIF-1α

Hypoxia-inducible factor-1α (HIF-1α) is a central regulator under hypoxic conditions, playing complex roles in SONFH pathogenesis.^144^ HIF-1α expression is oxygen-dependent, with levels increasing at 5% oxygen but decreasing at 1%.^145,146^ In general, GCs treatment disrupts the production of HIF-1α and thus inhibits HIF-1α/VEGF and Notch signaling pathways, which couple angiogenesis and osteogenesis.^147–149^ The HIF-1α/VEGF pathway also promotes alkaline phosphatase (ALP) and osterix expression while inhibiting PPARγ, thereby promoting bone remodeling and inhibiting adipogenesis (Fig. 6c).^145^ Interestingly, after the downregulation of the HIF-1α/VEGF pathway, the poor perfusion and hypoxic environment might lead to overexpression of HIF-1α, which can conversely mitigate the inhibitory effects of DEX on hypoxia-induced mitophagy and protect osteocytes from apoptosis.^150^ Of note, HIF-1α can stimulate IL-6 expression, a pro-inflammatory cytokine that contributes to synovitis during the later stages of SONFH (Fig. 6d).^151^ Also, by promoting RANKL expression, HIF-1α can contribute to osteoclastogenesis.^152–154^ Thus, it can bidirectionally influence bone formation and thus SONFH progression by interacting with multiple molecular factors (Fig. 6e).^145^

#### Coagulation-related factors

Dysregulation of blood clotting disrupts blood flow and compromises the blood supply to the femoral head, contributing to the pathogenesis of ONFH.^116^ Key factors implicated in this process include von Willebrand factor (vWF),^28^ tissue factor (TF), fibrinogen (FGA), and other thrombophilia-related proteins. GCs are found to increase the levels of procoagulant factors such as factor VIII and von Willebrand factor (VWF).^155^ Furthermore, GCs reduce tissue plasminogen activator (t-PA) and plasminogen activator inhibitor-1 (PAI-1) levels, potentially stimulated by elevated TNF-α.^98^ Factor V Leiden, a genetic risk factor for venous thrombosis caused by the G1691A mutation in thrombophilia factor V, was associated specifically with SONFH.^156^ In addition, elevated serum levels of S100A9, which promotes erythrocyte adhesion to vessel walls and thrombus formation, have been observed in SONFH patients.^98^ Immunoreactivity of S100A9 was particularly strong in thrombosed vessels, fibrotic tissue, osteocytes, and inflammatory cells within osteonecrotic lesions (Fig. 6f).^28,98^ Moreover, intravascular sickling and elevated hematocrit levels are additional risk factors for SONFH, as they can trigger thrombophilia and hypofibrinolytic coagulation abnormalities. However, not all sickle cell patients experience the complication, suggesting variability in individual susceptibility.^157,158^

### Signaling pathway cascades

#### TGF-β/Smad pathway

The transforming growth factor beta (TGF-β)/small mothers against decapentaplegic (Smad) pathway, also known as the canonical TGF-β pathway, plays a critical role in mediating cell proliferation, differentiation, migration, and apoptosis. Dysregulation of this pathway has been implicated in a variety of diseases, including cancer, fibrosis, and developmental disorders.^159–161^ The TGF-β protein family is synthesized as large precursor molecules comprising mature TGF-β and latency-associated protein (LAP). Activation of TGF-β, which depends on LAP cleavage by osteoclasts, establishes a gradient of active TGF-β that recruits BMSCs to facilitate osteogenesis during bone remodeling.^159–161^ Generally, GCs have been recognized to promote the activation of TGF-β signals in bones.^162,163^ Nevertheless, GCs have also been revealed to disrupt the Smad proteins to inhibit the TGF-β/Smad pathway.^164^ General control non-derepressible 5 (GCN5), as a downstream factor of Smad4, is significantly diminished in hBMSCs exposed to DEX. Silencing Smad4 effectively reverses the protective effects conferred by GCN5 overexpression.^165^ Interestingly, TGF-β/Smad signaling plays complex and sometimes contradictory roles in SONFH. On the one hand, TGF-β exerts protective effects in early ONFH, primarily by promoting osteoblast differentiation, maintaining bone homeostasis, and facilitating angiogenesis.^59,164,166,167^ On the other hand, reducing this pathway via Smad7 activation or interfering with Smad2/3 results in improved bone regeneration outcomes.^168–170^ Both insufficient TGF-β signaling and excessive activation of the pathway can possibly exacerbate SONFH.^159^ Thus, the role of TGF-β in SONFH is highly temporal and spatially regulated.

#### The Wnt/β-catenin pathway

**Fig. 7:**
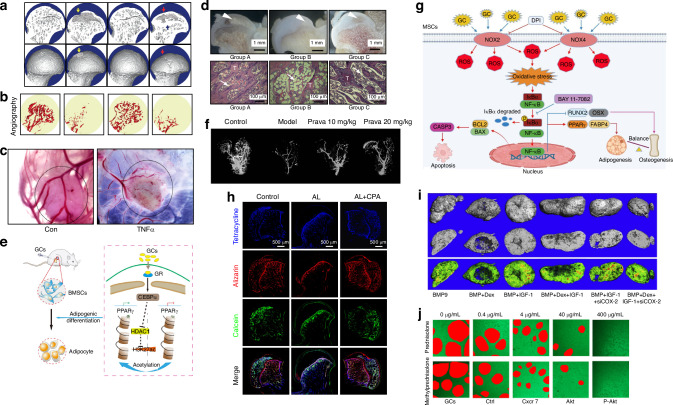
Signal pathway cascades dysregulation in SONFH. **a** Activation of CB2 attenuates GC-induced bone infarction and maintains femoral-head morphology in the SONFH rat model. Surface and profile view of femoral heads is shown. Red arrows: cortical fracture; yellow arrows: cortical disruption; blue arrows: compensatory sclerosis. Reproduced with permission. Copyright 2021, Sun et al.^177^ **b** Angiography images of femoral heads analyzed with micro-CT showing the effect of elevation of CB2. Reproduced with permission. Copyright 2021, Sun et al.^177^ **c** TNF-α promotes rMSCs proliferation and angiogenesis. The figure is the result of a 12-day-long PBS or TNF-α (10 ng/mL) loading into gelatin sponge and transplanted on the chick CAM. Reproduced with permission. Copyright 2019, Fang et al.^179^ **d** Macropathology (upper) and H-E staining (lower) coronal sections through the teres ligament of the femoral head. Showing that the inhibitor of P-gp would decrease the size of the EOC, the number and thickness of trabeculae (T), and increase the separation of trabeculae as well as the size and number of adipocytes (Group B) compared to the promoter group (Group A) and control group (Group C). Reproduced with permission. Copyright 2010, Springer Science Business Media.^181^ **e** A suggested mechanism on C/EBPα promoting adipogenic differentiation of BMSCs by targeting the PPARγ signaling pathway, while elevation of C/EBPα significantly impaired osteogenic differentiation. Histone H3K27 acetylation of PPARγ was also demonstrated to play an important role in the epigenetic mechanism underlying SONFH. Reproduced with permission. Copyright 2022, Duan et al.^186^ **f** Representative images of micro-CT reconstructed 3-D microangiography of proximal femur from control, model, Pravastatin 10 mg/kg, and Pravastatin 20 mg/kg groups showing that Pravastatin administration enhanced femoral head neovascularization. Reproduced with permission. Copyright 2014, the Society for Experimental Biology and Medicine.^187^ **g** Schematic diagram of the pathogenesis of SONFH. The aggravation of the OS rich microenvironment in MSCs induced by high doses of GCs, leading to apoptosis and differentiation imbalance, was a crucial factor in the pathogenesis of SONFH, which was mediated by activation of the NOX/ROS/NF-κB signaling pathway. Reproduced with permission. Copyright 2023, Xu et al.^125^ **h** CPA demonstrated protective effects against alcohol-induced ONFH. Fluorochrome labeling revealed a notable reduction in new bone formation in the alcohol-treated group. However, treatment with CPA significantly restored the capacity for bone formation, highlighting its pharmacotherapeutic potential. Reproduced with permission. Copyright 2020, Yu et al.^204^ **i** Effects of IGF-1, COX-2, and Dex on BMP9-induced ectopic bone formation. Micro-CT results showed the effect of IGF-1, COX-2, and Dex on BMP9-induced bone masses, bone density, and bone trabecular. Reproduced with permission. Copyright 2019, Elsevier.^205^ **j** Representative images of tube formation assay showing that GCs downregulated CXCR7 and impaired the angiogenesis function of EPCs in vitro. Reproduced with permission. Copyright 2019, Ding et al.^208^

The Wnt/β-catenin pathway, also known as the canonical Wnt pathway, is a vital signaling cascade involved in embryonic development and tissue homeostasis. The canonical Wnt/β-catenin pathway consists of four key segments: the extracellular signal, membrane segment, cytoplasmic segment, and nuclear segment. Extracellular signals are primarily mediated by Wnt proteins, such as Wnt3a, Wnt1, and Wnt5a. The membrane segment involves receptors, including Frizzled (a sevenfold transmembrane receptor protein) and LRP5/6. The cytoplasmic segment includes β-catenin, glycogen synthase kinase-3β (GSK-3β), and casein kinase I (CK1). Within the nuclear segment, β-catenin translocates to the nucleus to exert its bone recovery effects.^171^ The non-canonical pathway also promotes osteogenesis via suppressing PPAR-γ while elevating Runx2.^172,173^ GC is commonly known as an inhibitor of the Wnt/β-catenin pathway. Dickkopf 1 (DKK1), which is found to inhibit Wnt signal, is found to be elevated by GCs.^173^ Polydatin has been proven to rescue SONFH through inhibiting the promotion of DKK1 by GCs.^174^ GCs can also promote GSK-3β expression as a potential contributor to SONFH.^175^ Lithium chloride is a common inhibitor of GSK-3β whose administration has been shown to mitigate GC-induced bone loss.^176^ On the other hand, Wnt/β-catenin pathway activation has been identified as a potential therapeutic strategy for SONFH. Selective activation of cannabinoid receptor 2 (CB2), which is downregulated in necrotic femoral heads, provides relief for SONFH by upregulating Wnt/β-catenin signaling (Fig. 7a). CB2 activation also increases migration and tube-forming capacity in HUVECs (Fig. 7b).^177^

#### BMP/Runx2 pathway

BMPs, particularly BMP-2 and BMP-4, play critical roles in promoting osteoblast differentiation and bone formation by activating downstream Runx2 expression. Lower BMP-2 levels have been observed in necrotic femoral heads,^140^ while increased BMP-2 expression has been identified in BMSC-treated ONFH.^141^ However, Tingart et al.^32^ reported elevated BMP-2 and BMP-7 gene expression in the femoral head and neck regions of SONFH patients compared to those with osteoarthritis. Increased extracellular osteocalcin deposition in SONFH tissue, likely due to higher osteoblast counts and Runx2 activity, further supports the repairing state observed in SONFH. Excessive GCs dosage is recognized as a direct negative regulator of BMP2.^83,178^ Moreover, GC-induced TNF-α also fosters cell proliferation and angiogenesis (Fig. 7c) but hampers osteogenesis by increasing CpG methylation in the Runx2 promoter.^179^ As a GC receptor modulator, P-glycoprotein (P-gp) reduces SONFH risk by enhancing Runx2 expression via the BMP/Runx2 pathway (Fig. 7d).^180–182^ Similarly, ginsenoside Rb1 mitigates osteoblast dysfunction by upregulating BMP-2 expression and thus ameliorating SONFH.^183,184^

#### PPARγ pathway

Peroxisome proliferator-activated receptor-γ (PPARγ) is a nuclear receptor primarily expressed in adipose tissue, where it regulates adipocyte differentiation, fatty acid uptake, and lipid synthesis. Activation of the PPARγ pathway promotes the expression of adipogenesis-related genes, contributing to fat cell formation and lipid accumulation. Increased expression levels of PPARγ have been observed in SONFH patients,^120,125,185^ which reduces osteogenic activity, as evidenced by decreased osteocalcin levels and diminished ALP activity.^120^ It has been reported that C/EBPα preserves PPARγ activity and accelerates SONFH progression (Fig. 7e).^186^ Research has also demonstrated that suppressing PPARγ activity effectively reduces SONFH risk. For example, pravastatin regulates lipid levels by inhibiting PPARγ while simultaneously activating the Wnt/β-catenin signaling pathway, thereby promoting neovascularization and osteogenesis (Fig. 7f).^187^

#### NF-κB signaling pathway

The NF-κB pathway regulates inflammation, immune response, cell survival, and differentiation. It is activated by diverse extracellular signals such as pro-inflammatory cytokines, microbial products, and stress signals. GCs, which are believed to inhibit NF-κB to suppress inflammation, however, is nowadays found to be related to NF-κB elevation, prolonging osteoclast lifespan, and exacerbating SONFH. The NF-κB signaling pathway has been proven to be upregulated in SONFH, mediating oxidative stress and osteogenesis/adipogenesis balance (Fig. 7g).^125^ Liu et al.^188^ reported diminished inhibitor of NF-κB kinase epsilon (IKKe) levels in SONFH patients, and IKKe knockout models demonstrated enhanced osteoclastogenesis, reduced SONFH progression in murine models.

#### PI3K/Akt-related signaling pathways

The PI3K/Akt pathway is regarded as a central signaling hub, orchestrating numerous downstream pathways. Its versatility plays a pivotal role in cell survival, proliferation, and differentiation. A marked downregulation of p-Akt expression has been observed in the femoral heads of SONFH patients.^189^ Similarly, fibroblast growth factor 2 (FGF-2), an upstream promoting factor of PI3K/Akt pathway, is downregulated in DEX-treated osteoblasts.^190,191^ GC-induced PI3K/Akt pathway dysfunction is further implicated in ROS overproduction, which induces oxidative stress and apoptosis, exacerbating SONFH pathology.^192–199^ By contrast, activation of the PI3K/Akt pathway in osteoblasts promotes cellular proliferation, reduces ROS, and enhances osteogenic gene expression while decreasing RANKL levels.^200–203^ Chrysophanic acid (CPA) has been reported to counteract the suppression of the PI3K/Akt pathway, thus ameliorating ONFH (Fig. 7h).^204^ IGF-1, a PI3K/Akt agonist, reverses FOXO1 activation and alleviates SONFH phenotypes (Fig. 7i).^205^ PI3K/Akt pathway has also been found to interconnect with the Wnt/β-catenin pathway. Downregulation of the PI3K/Akt pathway activates GSK-3β, which contributes to DEX-induced osteoblast apoptosis and SONFH progression.^206,207^ On the other hand, Akt pathway downregulation in EPCs is also responsible for inhibited angiogenesis during SONFH progression (Fig. 7j).^208^

#### The JAK/STAT pathway

The Janus kinase/signal transducer and activator of transcription (JAK/STAT) pathway mainly transduces extracellular signals, such as cytokines and growth factors, generally inducing inflammatory lesions.^209,210^ In SONFH, the activated JAK/STAT pathway has been implicated in disease progression.^74,211^ Inhibition of the JAK/STAT1/3 pathway with Ruxolitinib has been shown to mitigate SONFH.^74^ Additionally, IL-21, another cytokine elevated in SONFH, contributes to cartilage inflammation and degradation through activation of the JAK/STAT1 signaling pathway.^212,213^ Furthermore, IFN-γ has been shown to activate the JAK1/2-STAT1 pathway, leading to M1 macrophage polarization, which is associated with a pro-inflammatory microenvironment within SONFH.^213^

### Genetic variations

**Fig. 8:**
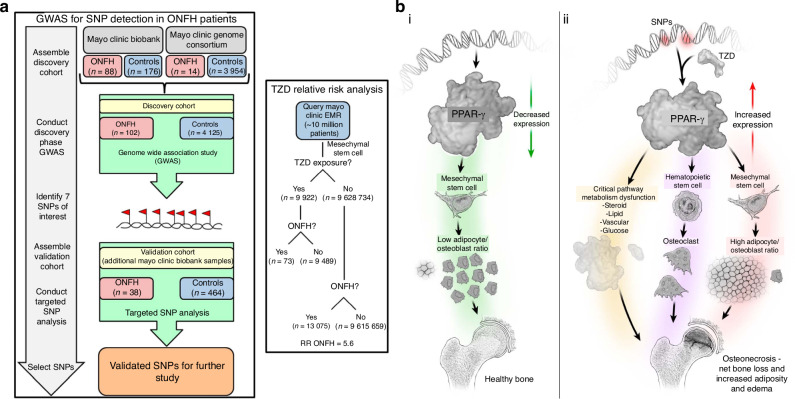
Genetic factors in SONFH. **a** The establishment of the discovery and validation cohorts for genetic assessment, along with the complementary pharmacologic impact data from the patient record. Reproduced with permission. Copyright 2019, the Association of Bone and Joint Surgeons.^215^ **b** Suggested mechanisms for PPARG involvement in the pathophysiology of ONFH are shown. (i) PPARG acts in a healthy state with low levels of expression, supporting the formation of normal bone alignment. (ii) Pathologic enhancement of the expression of PPARG, either through SNPs or pharmacologic modulation with TZD drugs, alters pathways resulting in osteonecrosis in bone. Reproduced with permission. Copyright 2019, the Association of Bone and Joint Surgeons.^215^

Gene polymorphisms have been implicated in the development of ONFH. Studies investigating the related gene variations in SONFH are listed in Table 5. Recent retrospective genome-wide association studies (GWAS) have emerged as essential tools for uncovering genetic contributions to musculoskeletal pathologies.^214^ A GWAS involving 140 non-traumatic SONFH patients and 4 589 controls (including 1 001 individuals with steroid exposure but no SONFH) identified seven tightly clustered single-nucleotide polymorphisms (SNPs) near the 3′ end of PPARγ as potential contributors to ONFH (Fig. 8a).^214,215^ These SNPs map to a region within a highly conserved area containing critical transcription factor binding sites. Polymorphisms in this region may disrupt three-dimensional chromatin organization, affecting interactions between the PPARγ 3′ end and its 5′ promoter and transcription start site.^214^ Additionally, targeted pharmacosurveillance involving 9 638 296 patient records confirmed an increased relative risk of ONFH (OR = 5.6; 95% CI, 4.5–7.1) among patients exposed to thiazolidinediones, a class of PPARγ agonists. Upregulated PPARγinfluences steroid metabolism and vasculogenesis, both critical to SONFH development (Fig. 8b).^214,215^

**Table 5:** Studies investigating the related gene variations and non-coding RNAs in SONFH

Reference	Animals	Involved Genes/RNAs	Involved cells	Relating pathways	Evaluation methodology	Outcome
Grässel et al.^216^	/	MMP-2/MMP-9 genes	/	/	RT-PCR, ELISA	MMP-2 was obviously elevated in SONFH patients whereas the upregulation of MMP-9 was not that significant.
Li et al.^217^	/	MMP-9 gene	/	/	ELISA, clinical scores	Increased MMP-9 expression and imbalance in the MMP-9/TIMP-1 ratio played a pivotal role in the development of SONFH.
Tian et al.^218^	/	MMP-2/MMP-10 genes	/	/	Linkage disequilibrium pattern	MMP-2 and MMP-10 polymorphisms were reported to be associated with SONFH.
Zhao et al.^219^	/	CR2 gene	/	/	Genetic association analyses	Polymorphisms of CR2 gene was significantly related with SONFH.
Jin et al.^220^	/	IL-4 gene	/	/	Statistical analysis	The rs2243283 in the IL-4gene was potentially associated with the occurrence of SONFH.
Liu et al.^239^	Rats	MiR-23b-3p	/	/	qRT-PCR, luciferase reporter assay	MiR-23b-3p played a protective role in SONFH by targeting ZNF667.
Li et al.^249^	Rats	MiR-672-5p, miR-146a-5p	/	/	qRT-PCR, microarray assay	Upregulated miR-672-5p expression and downregulated miR-146a-5p expression were significant after the GC administration.
Meng et al.^231^	Rats	MiR-141	BMSCs	/	Flow cytometry, RT-PCR	MiR-141 inhibited the proliferation of BMSCs in SONFH by targeting SOX11.
Xue et al.^232^	Rats	MiR-141	BMSCs	/	Luciferase reporter assays, qRT-PCR	MiR-141 inhibited the osteogenesis procedure of BMSCs in SONFH by targeting E2F3.
Zhang et al.^233^	/	MiR-93-5p/	BMSCs	/	Micro-CT, immunohistochemistry, qRT-PCR	MiR-93-5p in traumatic ONFH (TONFH) patients inhibited osteogenic differentiation via BMP-2 reduction.
Liu et al.^234^	/	MiR-93-5p miR-320a	/	/	Microarray assay, qRT-PCR	The expression of miR-93-5p and miR-320a in the serum of patients with TONFH were significantly elevated.
Zhang et al.^235^	/	MiR-320a-5p	BMSCs	/	qRT-PCR, immunohistochemistry, luciferase reporter assay	MiR-320a inhibited osteoblast differentiation via downregulating Runx2.
Xie et al.^240^	/	MiR-181d	BMSCs	TGF-β/Smad pathway	qRT-PCR, histology, luciferase reporter assay	MiR-181d could inhibit the differentiation of BMSCs into osteoblasts by negatively regulating Smad3.
Fu et al.^241^	/	MiR-596	BMSCs	TGF-β/Smad pathway	qRT-PCR, luciferase reporter assay	MiR-596 could suppress GC-induced BMSC osteoblastic differentiation by inhibiting Smad3.
Yang et al.^242^	/	MiR-100-5p	BMSCs, HUVECs	BMPR2/Smad1/5/9 pathway	qRT-PCR, luciferase reporter assay, histology, tube formation assay	MiR-100-5p could BMSCs and HUVECs damage by targeting BMPR2 and suppressing the BMPR2/Smad1/5/9 pathway.
Cao et al.^243^	Rats	MiR-224	BMSCs	Smad4-Taz pathway	qRT-PCR, luciferase reporter assay, histology	Enhancement of the expression of miR-224 was responsible for the GC-induced elevation of adipogenesis via the Smad4-Taz pathway.
Zhang et al.^244^	/	MiR-3137, MiR-340-3p	/	/	Weighted gene coexpression network analysis (WGCNA), machine learning algorithms	GYPA, TMCC2, and BPGM were main biomarkers for SONFH while GYPA might be regulated by hsa-miR-3137 and that BPGM might be regulated by hsa-miR-340-3p.
Zhang et al.^245^	/	MiR-135b-5p	/	/	KEGG and GO	MiR-135b-5p might be identified as a biomarker for SONFH.
Tang et al.^248^	/	MiR-27a	BMSCs	PI3K/Akt/mTOR pathway	Microarray assay, qRT-PCR, luciferase reporter assay	MiR-27a was thought to relieve ONFH via activating the PI3K/Akt/mTOR pathway.
Wu et al.^247^	/	MiR-135b	BMECs, HUVECs	/	Micro-CT, immunohistochemistry	ESWT relieved endothelial injury through elevating the miR-135b targeting FOXO1.
Nan et al.^314^	Rats	MiR-146a	BMSCs	Wnt/FOXO pathway, Sirt1/NF-κB pathway	Micro-CT, immunohistochemistry, RT-PCR	Resveratrol (Res) prevented SONFH by upregulating miR-146a, and thus stabilizing osteogenesis/osteoclastogenesis homeostasis via Wnt/FOXO and Sirt1/NF-κB pathways.
Li et al.^249^	Mice	MiR-182-5p	BMSCs	MYD88/Run2 pathway	RT-PCR, wound-healing assay, micro-CT	MiR-182-5p regulated osteogenesis in the SONFH via targeting MYD88 and the following activation of Runx2.
Li et al.^246^	/	MiR-195-5p	/	/	RT-PCR	Collapse at the necrosis area might be related to the downregulation of miR-195-5p.
Wang et al.^251^	Rats	MiR‑196a‑5p	BMSCs	/	Gene chip assay, RT-PCR	The decrease in miR‑196a‑5p might be significant in SONFH.
Huang et al.^252^	Rats	MiR-148a-3p	BMSCs	SMURF1/Smad7/Bcl-2 pathway	RT-PCR, WB	MiR-148a-3p overexpressed in BMSC-EVs elevated the expression of Smad7 and BCL-2 via inhibiting SMURF.
Bai et al.^253^	Rats	MiR-27a	MC3T3‑E1 cells	TGF‑β/Smad7 pathway	Histology, WB	MiR-27a regulated SONFH through inducing TGF‑β/Smad7 pathway.
Luo et al.^254^	/	LncRNAs	/	/	Microarray, KEGG, and GO	The disorder of lncRNAs and mRNAs might resulted in the pathology of SONFH.
Wu et al.^256^	/	LncRNA FGD5-AS1	BMSCs	MiR-296-5p/STAT3 pathway	RT-PCR, WB	LncRNA FGD5-AS1 promoted cell proliferation by sponging miR-296-5p.
Wang et al.^257^	/	LncAABR07053481	BMSCs	MiR-664-2-5p/Notch1 pathway	Cluster analysis, immunofluorescence, histology	LncAABR07053481 inhibits hypoxia-induced apoptosis of BMSCs and demonstrated protective effect of SONFH by regulating the miR-664-2-5p/Notch1 pathway.
Zhang et al.^258^	/	LncRNA EPIC1	/	/	qRT-PCR	Lnc-EPIC1 protected osteoblasts from Dex via regulation of Myc.
Wang et al.^263^	/	LncRNA MALAT1 and HOTAIR	BMSCs	/	Histology, RT-PCR	LncRNA MALAT1 and HOTAIR were associated with aberrant osteogenic and adipogenic differentiation of BMSCs.
Li et al.^264^	/	LncRNA MAPT‑AS1	BMSCs	/	Histology, RT-PCR, KEGG, and GO	LncRNA MAPT‑AS1 was found to pivotally related to osteogenesis.
Chen et al.^260^	/	LncRNA-AWPPH	BMSCs	BMP/Runx2 pathway	RT-PCR	LncRNA-AWPPH could alleviate SONFH through enhancing Runx2.
Xiang et al.^265^	/	LncRNA RP11-154D6	BMSCs	/	qRT-PCR, immunohistochemistry	The abnormal downregulation of lncRNA RP11-154D6 in SONFH might be related to the inhibition of osteogenesis.
Jin et al.^262^	/	LncRNA MIAT	Adipose-derived stem cells (ASCs)	/	RT-PCR, micro-CT	MIAT was increased by the regulation of TNF-α and was related to the pro-inflammatory process.
Xiang et al.^266^	/	Circ_0000219, Circ_0005936	BMSCs	/	RT-PCR	hsa_circ_0000219 and hsa_circ_0005936 might regulate the progression of ONFH by mediating the proliferation and differentiation of BMSCs.
Chen et al.^267^	/	CDR1as	BMSCs	CDR1as-miR-7-5p-WNT5B pathway	RT-PCR, MRI, microarray assay	The elevation of CDR1as in SONFH models was demonstrated to inhibit osteogenesis through the CDR1as-miR-7-5p-WNT5B pathway.
Feng et al.^269^	/	CircHGF	BMSCs	miR-25-3p/Smad7 pathway	RT-PCR, microarray assay	CircHGF suppressed BMSCs proliferation via inhibiting the miR-25-3p biding to Smad7.
Han et al.^270^	Rats	Circ_0058792	MC3T3-E1 cells	miR-181a-5p/Smad7 pathway	RT-PCR, luciferase reporter assay	Circ_0058792 inhibited osteogenic differentiation by sponging miR-181a-5p via the TGF-β/Smad7 pathway.
Hao et al.^168^	Rats	CircPVT1	BMSCs	TGF-β/Smad7 pathway	Histology, RT-PCR, luciferase reporter assay	CircPVT1 regulated SONFH via modulating miR-21-5p/ Smad7/TGFβ pathway

Matrix metalloproteinases (MMPs), responsible for ECM remodeling, are also implicated in ONFH.^216,217^ Polymorphisms in MMP-2 and MMP-10 were specifically associated with SONFH susceptibility in the Chinese Han population. Genetic analysis identified six significant SNPs (rs470154, rs243866, rs243864, rs865094, rs11646643, and rs2241146), with rs2241146 (MMP-2) and rs470154 (MMP-10) demonstrating statistically significant associations with increased SONFH risk.^218^

Polymorphisms in genes related to B-cell differentiation and immunoglobulin production, such as the CR2 gene, increase SONFH risk.^219^ Additionally, polymorphisms in the IL-4 gene influence its anti-apoptotic capabilities, with rs2243283 reducing IL-4 function, while rs2243289 is protective against SONFH.^220^ SNPs of IL-6 also affect its functions in the pathology of SONFH.^221,222^ Rs2069837 G>A polymorphism in the IL-6 gene was significantly associated with a decreased risk of ONFH among the Chinese Hans.^223^ Moreover, a Chinese case-control comparison and subsequent meta-analysis revealed that promotion of polymorphisms TNF-α-238G>A and -308G>A are significantly linked to increased susceptibility to non-traumatic ONFH. The -308A variant of TNF-α has also been reported that when combined with hypoxia, it escalates the risk of SONFH, suggesting a gene-environment interaction.^179,224,225^

Epigenetic mechanisms have also been crucial to the pathology of SONFH, mainly including DNA methylation and histone modifications. Firstly, genome-wide and candidate gene studies have identified hypermethylation of FZD1, a Wnt receptor, leading to Wnt/β catenin pathway suppression in BMSCs from SONFH patients.^226,227^ Besides, m6A modification, mediated by METTL14, was found to be reduced in SONFH patients. Decreased m6A levels result in the degradation of PTPN6 mRNA, a GSK-3β inhibitor, further suppressing the Wnt pathway.^228^ Alterations in 5-hydroxymethylcytosine (5hmC), mediated by the TET enzyme family, may also promote osteocyte apoptosis and may exacerbate SONFH progression.^229^ In addition, dysregulated histone deacetylases, particularly SIRT1, SIRT3, and HDAC9, have been implicated to be associated with SONFH, since GCs downregulate these enzymes and induce cell apoptosis.^130,230^ Of note, the decrease in HDAC91 induces the elevation of the acetylation level of histone H3K27 in the PPARγ promoter region, resulting in promoted adipogenesis.^186^

### Non-coding RNAs

Non-coding RNAs (ncRNAs) refer to functional RNA molecules that do not translate into a protein. Small RNAs like microRNAs (miRNAs), circular RNAs (circRNAs), and long ncRNAs (lncRNAs) were found to play a critical role in the pathogenesis of SONFH. Studies investigating the related non-coding RNAs in SONFH are shown in Table 5.

#### MicroRNAs (miRNAs)

Acting as post-transcriptional regulators by binding to target mRNAs, miRNAs either promote mRNA degradation or inhibit mRNA translation. This regulatory mechanism allows miRNAs to function as critical modulators of signaling pathways and gene expression. Numerous studies have highlighted the pivotal role of miRNA-mediated gene expression in the onset and progression of SONFH.

**Fig. 9:**
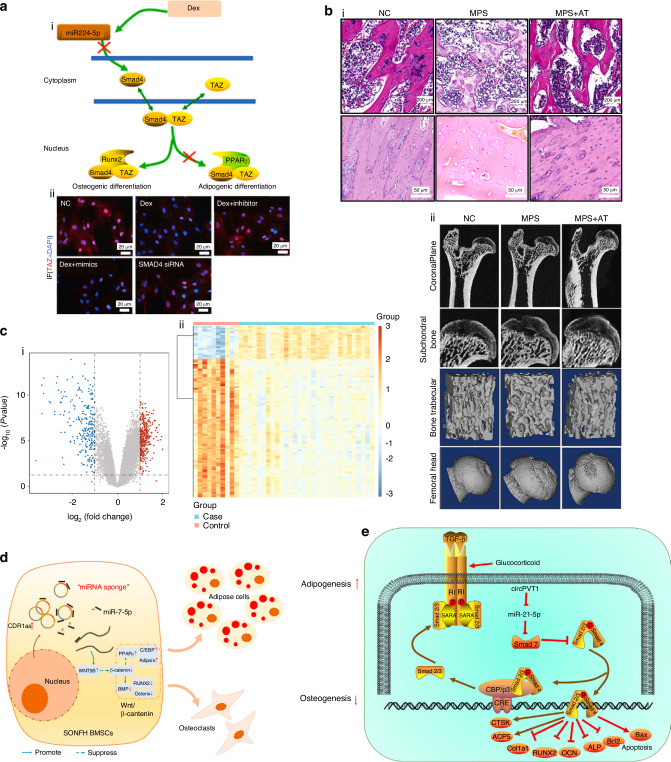
Non-coding RNAs regulating the process of SONFH. **a** MiR-224-5p regulates the nuclear retention of TAZ. (i) shows a schematic diagram of the TAZ mechanism. The Smad4-TAZ axis was supposed to play a pivotal role in adipogenesis and osteogenesis because TAZ in the nucleus could interact with both Runx2 and PPARγ. (ii) shows the immunofluorescence examination of the nuclear retention of TAZ. Reproduced with permission. Copyright 2021, Elsevier.^243^ **b** (i) Histological analysis of the femoral head revealed a distinct characteristic in the methylprednisolone group: empty bone depressions and necrosis with bone cell adhesion. Remarkably, treatment with miR-224-5p antagomir significantly reduced the occurrence of osteonecrosis of the femoral head from 58.3% to 8.3%. (ii) Micro-CT result showed that MiR-224-5p antagomir could significantly increase the trabecular thickness while decreasing the trabecular separation of the femoral head. Reproduced with permission. Copyright 2021, Elsevier.^243^ **c** DEGs between SONFH patients and control samples. Volcano plot (i) showing the expression levels of DEGs. Heat map (ii) showing the expression levels of the top 100 DEGs. Reproduced with permission. Copyright 2022, Zhang et al.^244^ **d** The mechanism of the regulatory effect of CDR1as in SONFH-BMSCs. Reproduced with permission. Copyright 2020, Chen et al.^267^ **e** The mechanistic illustration shows that in SONFH, CircPVT1 is decreased. Elevating CircPVT1 levels results in decreased expression of TGFβ/Smad2/3 and enhances Smad7 activation through targeting miR-21-5p, consequently reducing SONFN. Reproduced with permission. Copyright 2021, Hao et al.^168^

Upregulated miRNAs often act by either facilitating adipogenesis, inhibiting osteogenesis, or promoting inflammatory and oxidative stress responses. MiR-141 inhibits BMSCs' proliferation in SONFH by targeting SOX11, which enhances BMSCs' proliferation.^231,232^ Similarly, miR-141 targets E2F transcription factor 3 (E2F3), and its knockdown enhances E2F3 expression, restoring osteogenesis in BMSCs.^232^ GC-induced miR-93-5p negatively regulates BMP2 expression, impacting osteoblast proliferation,^233,234^ while miR-320a-5p directly inhibits Runx2, promoting adipogenesis and suppressing osteogenic markers like osterix, collagen I, and osteocalcin.^235^ In the adipogenesis process, miR-148a is enriched in adipocyte-derived microvesicles of SONFH patients, enhancing adipogenesis and suppressing osteogenesis.^236^ Upregulation of miR-672-5p after GCs treatment was shown to target Angptl4, inhibiting angiogenesis and promoting adipogenic differentiation.^237^ Moreover, GC-induced miR-206 leads to osteoclast differentiation while suppressing osteoblast proliferation.^238^ Overexpression of miR-23b-3p in a rat SONFH model led to reduced plasma viscosity, lower blood lipid and pro-inflammatory cytokine levels, and improved bone integrity.^239^ The Smads family is a common downstream target of miRNAs. GC-induced miR-181d and miR-596 suppress Smad3 expression, inhibiting osteoblast differentiation.^240,241^ Elevated miR-100-5p in exosomes from SONFH patients targets BMPR2, impairing the BMPR2/Smad1/5/9 pathway, which exacerbates angiogenesis and osteogenesis defects.^242^ MiR-224-5p, upregulated in GCs-treated BMSCs, inhibits the Smad4-TAZ pathway, downregulating Runx2 and enhancing PPARγ activity, thereby aggravating SONFH (Fig. 9a).^243^ Moreover, inhibition of miR-224-5p has been shown to reverse GC-induced effects and promote bone formation in SONFH models (Fig. 9b).^243^ Additional mechanisms include miR-335, which downregulates eNOS and SOD, increasing platelet aggregation and ROS levels.^9^ Upregulated miR-137-3p inhibits the CXCL12/SDF-1α pathway, reducing EPC mobility, while miR-132-3p repairs BMEC damage.^9^ Weighted gene coexpression network analysis (WGCNA) identified downregulation of GYPA and BPGM in SONFH, with GYPA regulated by hsa-miR-3137 and BPGM by hsa-miR-340-3p (Fig. 9c).^244^ Additionally, sequencing data revealed upregulated hsa-miR-135b-5p in SONFH, although its exact role remains unclear.^245^

Downregulated miRNAs generally contribute to the progression of SONFH by impairing osteogenesis and promoting inflammatory processes. In SONFH patients, reduced levels of miR-182-5p in extracellular vesicles (EVs) directly activate pro-inflammatory pathways, while inhibition of these pathways delays SONFH progression.^246^ Downregulation of miR-146a-5p induced by GCs also leads to pro-inflammatory and pain.^237^ Nan et al.^240^ demonstrated that miR-146a modulates osteogenesis/osteoclastogenesis homeostasis, thereby exerting a protective role against SONFH. However, in endothelial cells, miR-135b downregulation was associated with FOXO1 activation induced by DEX treatment, contributing to SONFH progression.^247^ Similarly, miR-27a, which is reduced following GCs treatment, has been implicated in alleviating SONFH by targeting the PI3K/Akt/mTOR pathway and reversing GC-induced repression of osteogenic differentiation in hBMSCs.^248^ Furthermore, miR-195-5p is significantly reduced in the collapsed region, leading to disrupted osteoblast proliferation, increased osteocyte apoptosis, and subsequent structural collapse.^249^ Restoring miRNA expression levels can mitigate SONFH pathogenesis. For instance, upregulating miR-410 has been shown to enhance osteoblast numbers while reducing osteoclasts, thereby alleviating SONFH.^250^ In BMSCs from a rat SONFH model, bioinformatics analysis revealed that downregulated miR-148a-3p decreases Smad7 ubiquitination and degradation, enhancing the Smad7/Bcl-2 pathway.^251,252^ Additionally, miR-27a downregulation in SONFH models was found to impair BMP-2 and Runx2 expression while increasing caspase-3/9 activity and Bax expression.^253^

#### LncRNAs

Long non-coding RNAs (lncRNAs) play a crucial regulatory role in the development of SONFH by modulating miRNA and mRNA expression through the lncRNA-miRNA-mRNA axis. Differential expression of lncRNAs in SONFH patients has been linked to cellular apoptosis, osteogenesis, adipogenesis, and inflammation. Luo et al.^254^ conducted the first comprehensive microarray analysis of lncRNA expression profiles in SONFH, revealing significant upregulation of immune system-related processes. Li et al.^255^ further identified that lncRNA FGD5-AS1 inhibits apoptosis in DEX-treated hBMSCs by sponging miR-296-5p and upregulating STAT3, thereby promoting cell survival.^256^ LncAABR07053481 mitigates GC-induced hypoxia and apoptosis in BMSCs by alleviating the repression of miR-664-2-5p on Notch1, improving BMSCs' survival, and enhancing reparative effects in osteonecrotic areas.^257^ EPIC1, an epigenetically-induced lncRNA, interacts with Myc to reduce GC-induced apoptosis, providing a repair mechanism during SONFH progression.^258^

LncRNAs have also been extensively studied in the regulation of osteogenic and adipogenic differentiation of BMSCs. HOTAIR sponges miR-17-5p, inhibiting Smad7 expression and subsequently downregulating BMP2, which worsens SONFH. It also targets miR-122, reducing PPARγ inhibition and promoting adipogenesis. In contrast, MALAT1 activates the AMPK pathway by inhibiting PPM1E and upregulating the antioxidant activity of Nrf2.^259^ It also sponges the miR-214/ATF4 pathway, enhancing osteogenesis and protecting against SONFH. MAPT AS1, another lncRNA, promotes osteogenesis while inhibiting adipogenesis, providing protective effects against SONFH.^255^ LncRNA-AWPPH is decreased in SONFH and has been shown to elevate Runx2 expression, promoting osteogenesis and serving as a potential therapeutic target for SONFH. Similarly, RP11-154D6 alleviates SONFH by reducing adipogenesis and suppressing PPARγ. Myocardial infarction-associated transcript (MIAT) silencing promotes osteogenesis, increases VEGF expression by targeting miR-200a, and facilitates BMSCs differentiation into endothelial cells^260–262^

LncRNAs also play a critical role in inflammatory responses and their contribution to SONFH. A regulatory network of 67 lncRNAs, acting downstream of the hsa-miR-320a/NOD2 pathway, was identified to participate in inflammatory reactions that contribute to SONFH progression, underscoring the importance of lncRNAs as both biomarkers and potential therapeutic targets in SONFH.^256–258,260,262–265^

#### Circular RNAs (circRNAs)

CircRNAs modulate downstream signaling pathways by acting as molecular sponges for specific miRNAs. Xiang et al.^266^ identified 231 differentially expressed circRNAs through sequencing in SONFH patients and controls. Among the identified circRNAs, hsa-circ-0000219, hsa-circ-0004588, and hsa-circ-0005936 were significantly reduced in SONFH patients. These circRNAs showed increased expression during osteogenic differentiation and decreased expression during adipogenic differentiation of in vitro induced BMSCs. The downstream targets of these circRNAs, such as miR‑144‑3p and miR‑1270, were confirmed to be silenced in SONFH.

CircRNA CDR1as has been shown to play a critical role in the disruption of adipogenic and osteogenic differentiation of BMSCs in SONFH. Acting as a sponge for miR-7-5p, CDR1as promotes the expression of Wnt5B, leading to decreased β-catenin activity. This interaction disrupts the Wnt signaling pathway, contributing to SONFH by impairing the balance between osteogenesis and adipogenesis (Fig. 9d).^267,268^ Similarly, circHGF, found to be upregulated in SONFH, interacts with miR-15-3p as a sponge, which reduces the inhibition of Smad7, thereby promoting osteogenesis and alleviating SONFH. In a similar manner, circ-0058792 interacts with miR-181a-5p, which targets Smad7. The upregulation of miR-181a-5p leads to increased phosphorylation of Smad2 and Smad3, further enhancing osteogenesis.^269,270^ Hao et al.^168^ reported that circPVT1 shares a similar regulatory relationship with miR-21-5p, influencing the TGF-β/Smad pathway in SONFH models (Fig. 9e). Broader network analyses have identified other circRNAs, such as hsa-circ-0000551, hsa-circ-0008928, and hsa-circ-0003915, which appear to regulate pathways implicated in SONFH. These circRNAs were highlighted in GO, KEGG, and PPI analyses for their potential involvement in SONFH pathology, although their specific roles in SONFH remain unclear.^271^ These findings suggest a complex network of circRNA-mediated regulation of critical pathways in SONFH.

## Discussion

Over the last decade, research into SONFH has made substantial progress, producing numerous promising findings. However, due to the multifactorial complexity of SONFH, a comprehensive summary of its pathogenic mechanisms remains elusive.

GCs have been shown to exert dose-dependent effects on the pathology of SONFH. In general, studies have demonstrated that steroid doses exceeding 20 mg prednisone per day significantly elevate the risk of ONFH, with every 10 mg/day increment during the first six months increasing the risk by approximately 4.6%.^17,272^ More specifically, low-dose GCs (10^−^^9^–10^−8 ^mol/L) have beneficial effects on the osteogenesis of BMSCs, whereas pharmacological doses (10^−^^7^–10^−^^6 ^mol/L) inhibit osteogenesis and promote an adipogenic shift.^87–92,273^ In vivo experiments also demonstrate that a dosage of 13.5 mg/kg/week of DEX and daily doses exceeding 7.5 mg of prednisolone equivalent are sufficient to suppress bone mass in mice.^273,274^ A similar dual effect is observed in the regulation of bone resorption. Five days of low-dose GC treatment (10^−8 ^mol/L) initially prolongs osteoclast survival via RANKL signaling, whereas pharmacological exposure (10^−7 ^mol/L) disrupts the cytoskeleton and reduces osteoclast precursor proliferation.^275,276^ In contrast to the dual effects on BMSC differentiation, GCs have consistently been reported to impair blood perfusion in SONFH.^17^ Studies have shown that in vitro exposure to 10^−6 ^mol/L GCs and in vivo administration of hydrocortisone at 100 mg/m^2^/day significantly inhibit angiogenesis of type H blood vessels.^277^

Patient heterogeneity also contributes to the complexity of SONFH pathology. Studies have investigated the impact of patient demographics on SONFH morbidity. Notably, the gender distribution of SONFH varies across countries. For example, while studies in the United States have reported a relatively even gender distribution, studies in China have revealed a much higher prevalence among males.^4,278,279^ Although large-cohort studies suggest a slight male predominance, females, who have a higher prevalence of autoimmune diseases such as systemic lupus erythematosus (SLE), are more frequently exposed to long-term GCs therapy. Moreover, differences in sex hormone levels, as well as their interactions with the immune system, have been considered contributory factors in the pathogenesis of SONFH.^280^ The pathogenesis, disease progression rate, and treatment responses of SONFH between men and women also remain to be further studied.

Most current investigations into the pathology of SONFH rely on animal models. As highlighted by Li et al.^281^ despite small animal models such as rabbits and rats being widely used due to their low cost and ease of handling, their skeletal structure, load-bearing characteristics, and bone metabolism differ significantly from humans. Specifically, the widespread use of quadruped models in SONFH research poses challenges, as their gait and hip biomechanics differ markedly from the human bipedal system, influencing lesion development, mechanical stress distribution, and collapse patterns.^281–285^ In addition, GCs' metabolism varies across species, leading to inconsistent SONFH induction rates (ranging from 15% to 90%) in rabbit and rat protocols.^283,284^ Furthermore, low reproducibility further interferes with model outcomes. Steroid-only induction often yields moderate SONFH incidence rates (approximately 33%–43%) in rabbits, whereas combining steroids with lipopolysaccharide (LPS) or allogeneic serum improves lesion consistency (up to 90%) but increases mortality.^25,283^ Potential enhancements in SONFH models may involve improved species selection, such as bipedal emu models,^281^ which exhibit hip joint anatomy and weight-bearing characteristics comparable to humans. Additionally, while combined-induction approaches have achieved higher induction rates, the dosage of endotoxins and timing of injections require further optimization to reduce mortality. Incorporating controlled mechanical loading or surgical vascular deprivation may also enhance pathological stress similar to that occurring in humans. Finally, employing comprehensive evaluation systems, including X-ray, CT, MRI, histopathology, and biomechanical testing, can further improve the reliability and translational relevance of SONFH animal model studies.

Diagnosis and prediction of SONFH have been greatly improved with the occurrence of artificial intelligence (AI). Machine learning algorithms like logistic regression (LASSO) and support vector machines-recursive feature elimination (SVM-RFE) integrate data from MRI or biomarkers, creating predictive and diagnostic models for faster and more accurate diagnosis of SONFH.^286–290^

In terms of therapeutic mechanisms, early-stage interventions, including physical therapies, pharmacological treatments, often yield suboptimal results.^2^ Core decompression (CD) may improve microcirculation, potentially leading to the release of factors such as VEGF and NO, but failed to induce necrotic bone reconstruction. As the disease progresses, total hip arthroplasty (THA) becomes the standard treatment, which serves as a more direct approach by replacing necrotic bone with a prosthesis.^9–11^ Other drugs targeting SONFH-related factors or pathways that are found effective in other related diseases, like HIF-1α, VEGF, Wnt/β-catenin, RANKL-NF-κB, and JAK/STAT, also show potential clinical value.^291–294^ Based on current knowledge, Wnt/β-catenin activation (Wnt activators, anti-sclerostin antibody, Romosozumab) and RANKL inhibition (Anti-RANKL antibody, Denosumab) represent the most promising therapeutic directions. HIF-1α (HIF stabilizers, Roxadustat) and IL-6 (IL-6R antibody, Tocilizumab) also show therapeutic potential and clinical drug availability. In contrast, PPARγ antagonism and direct VEGF delivery, while mechanistically relevant, face greater challenges in drug safety, targeting, or delivery efficacy. Novel treatment strategies have emerged, including traditional Chinese medicine,^295–305^ extracellular vesicle therapy,^306–315^ extracorporeal shock wave therapy,^316,317^ and pulsed electromagnetic field strategy.^318^ These therapies, combined with molecular insights, provide hope for innovative SONFH treatment development.

**Fig. 10:**
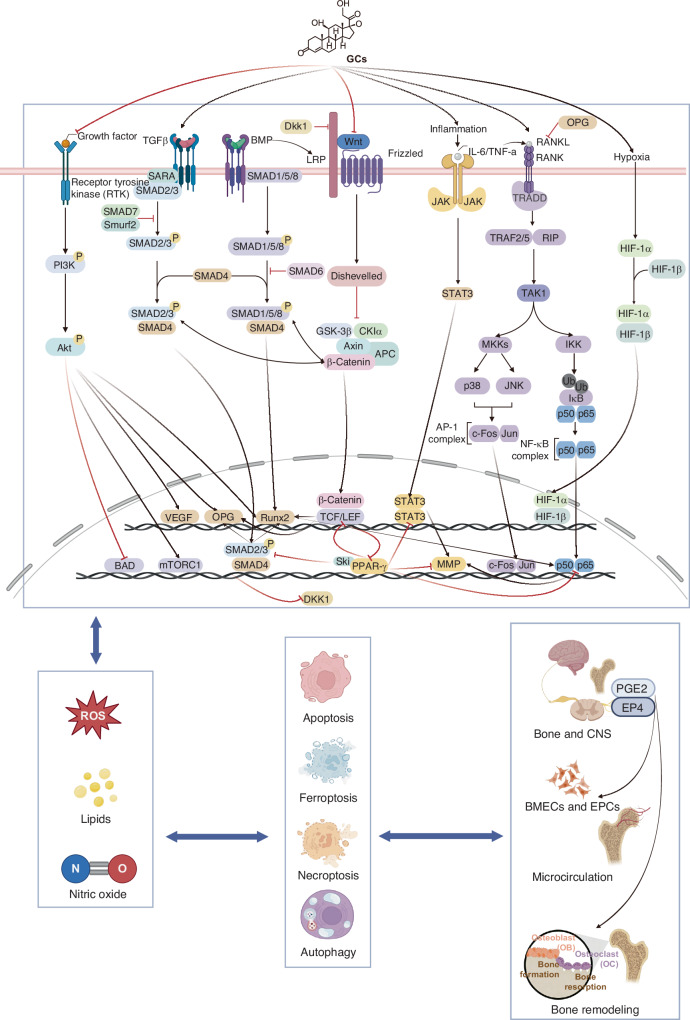
Schematic representation of the complex signaling network involved in SONFH. GCs' exposure modulates multiple signaling pathways, including TGF-β/SMAD, BMP/SMAD, Wnt/β-catenin, PI3K/Akt, JAK/STAT3, and RANKL/NF-κB pathways, affecting transcriptional regulation via PPARγ, MMPs, and downstream transcription factors (e.g., AP-1, NF-κB, TCF/LEF). These alterations lead to dysregulation of bone homeostasis, inflammation, and hypoxia responses, contributing to oxidative stress (ROS), lipid peroxidation, nitric oxide imbalance, and activation of cell death pathways such as apoptosis, ferroptosis, necroptosis, and impaired autophagy. The cumulative effects result in impaired microcirculation, CNS-bone axis dysfunction, and aberrant bone remodeling, ultimately driving SONFH progression

The intricacy of the pathology of SONFH also lies in the complicated crosstalk between risk factors, including impaired microcirculation, reduced bone formation, disordered metabolism, and a dysregulated immune system. For example, vascular injury (e.g., endothelial apoptosis, reduced angiogenesis via VEGF suppression) leads to hypoxia and impaired nutrient supply,^100,102,145,147–149^ which in turn promotes osteocyte and osteoblast apoptosis, exacerbating bone collapse.^24,25,319^ Moreover, GC-induced metabolic disorders, such as lipid accumulation and oxidative stress, not only compromise vascular integrity (e.g., via fat emboli and ROS-induced endothelial damage),^118–120,126,127^ but also shift BMSC differentiation toward adipogenesis while suppressing osteogenesis.^173,320^ Cell death mechanisms, including apoptosis, ferroptosis, and necroptosis across different cell types, are interconnected with oxidative and inflammatory signaling cascades (e.g., NF-κB, PI3K/Akt inhibition), many of which are initiated or amplified by GC exposure and vascular hypoperfusion, ultimately leading to reduced bone mass and microcirculatory dysfunction.^67–70,321,322^ Feedback loops involving neurovascular regulation,^323,324^ bone-CNS interoception, immune modulation, and even biomechanical alternations^325^ further reinforce the disease process. However, many of these interactions have not yet been fully elucidated in the context of SONFH pathology. At the molecular level, crosstalk between signaling pathways further weaves the complex network underlying SONFH pathogenesis. For instance, TGF-β/Smad pathway can facilitate β-catenin stabilization, thereby synergistically enhancing osteogenesis alongside Wnt/β-catenin signaling.^326,327^ TGF-β/Smad pathway can also interface with both Wnt/β-catenin and PI3K/Akt pathways to induce ROS generation, linking it to redox imbalance and ferroptosis.^164,328^ Interestingly, BMPs, members of the TGF-β superfamily, can both synergize with or inhibit Wnt/β-catenin signaling to bidirectional regulate osteoclast activation, suggesting environment-dependent interactions between pathways.^328,329^ The Wnt/β-catenin pathway, central to cell fate determination, not only integrates with TGF-β/Smad signaling but is also antagonized by PPARγ, which inhibits β-catenin nuclear localization. Moreover, Wnt signaling can be modulated by oxidative stress, with ROS promoting β-catenin degradation.^173^ The PI3K/Akt signaling pathway, reported to form a positive feedback loop with Wnt/β-catenin and to regulate NF-κB expression,^329,330^ also promotes PPARγ expression, thereby enhancing adipogenesis.^331^ NF-κB, a master regulator of inflammation and immune responses, is activated by ROS and further promotes the expression of pro-inflammatory genes.^320^ Other factors and pathways, including JAK/STAT, PGE2, VEGF, and NO, are also interwoven into a complex pathological network contributing to SONFH (Fig. 10).^320,323,324,332–334^

For SONFH progression, the same pathway may exert different or even opposing effects under varying circumstances. For instance, the TGF-β/Smad pathway can play contradictory roles in the progression of SONFH. On one hand, a gradient of active TGF-β released from the bone matrix ameliorates SONFH by spatially and temporally recruiting BMSCs to bone resorption sites and promoting bone formation during osteoclast-mediated resorption.^159^ However, prolonged high concentrations of active TGF-β can disrupt these chemotactic gradients and impair MSC targeting, which may explain its inhibitory effects on bone formation observed in certain SONFH models.^159,170^ Moreover, in the cell line of osteoblasts, TGF-β signaling in osteogenesis is stage-specific, showing distinct effects at different phases of osteoblast differentiation. During the early stages of osteoblast differentiation, TGF-β promotes the proliferation of osteoprogenitor cells and supports osteogenesis, whereas during the late stages, TGF-β signaling inhibits bone formation by negatively regulating ATF4, a key osteogenesis-related transcription factor.^328,335–339^ Similarly, NF-κB can facilitate bone remodeling in the early stages of SONFH, but its prolonged activation leads to inflammatory damage and bone loss.^320^

Gene polymorphisms can also synergistically influence the progression of SONFH. For instance, polymorphisms in PPARγ, MMP, and TNF-α can respectively enhance adipogenesis, extracellular matrix degradation, and inflammation, collectively exacerbating SONFH. Moreover, genes with SNPs can interact in complex patterns that dictate SONFH progression. For example, while PPARγ activation downregulates MMP-9, TNF, and interleukin genes, the upregulation of pro-inflammatory genes can drive MMP expression. PPARγ activation downregulates MMP-9 expression, primarily by inhibiting NF-κB signaling. This suggests a potential regulatory axis that might similarly mitigate matrix degradation in SONFH.^164,340–342^ For individuals with specific SNPs, clinicians might consider the early introduction of preventive therapies, such as PPARγ antagonists, anti-resorptive agents (e.g., bisphosphonates), or anti-inflammatory strategies. However, most current research has only established associations between SNPs and SONFH, with few studies clarifying whether these polymorphisms can reliably predict SONFH risk or whether reversing these gene alteration could prevent SONFH progression,^214,215,223^ highlighting an important direction for future research.

Given the multifactorial nature of SONFH pathogenesis, therapeutic strategies that simultaneously target vascular injury, metabolic dysfunction, and impaired bone remodeling may offer greater efficacy than monotherapies. For instance, early vascular damage and microthrombosis are central to femoral head ischemia, while GC-induced lipid accumulation further exacerbates bone loss. Therefore, combining anti-thrombotic or endothelial-protective agents with metabolic modulators may yield synergistic benefits. Statins, widely used for dyslipidemia, have shown promise in SONFH by improving endothelial function, reducing inflammatory cytokine levels, and inhibiting osteocyte apoptosis. Clinical studies have also reported a reduced risk of ONFH in patients receiving long-term statin therapy.^343,344^ Additionally, semaglutide, a GLP-1 receptor agonist primarily indicated for type 2 diabetes and weight management, has demonstrated vascular protective effects, anti-inflammatory properties, and beneficial modulation of lipid metabolism and bone turnover in preclinical studies.^345^ Its ability to reduce visceral fat while potentially enhancing bone quality makes it a compelling candidate for SONFH treatment.

From translation perspective, several challenges hinder preclinical therapies into clinical practice. First, current animal models cannot fully replicate the anatomy and biomechanics of the human femoral head. Second, the clinical complexity of SONFH, which often occurs in patients with autoimmune diseases or immunosuppression, making it more difficult for preclinical animal models to mimic the pathophysiological conditions. Third, many candidate compounds lack comprehensive pharmacological data. For example, resveratrol,^346^ icariin,^347,348^ and astragaloside IV^62,189^ have shown promising effects in cell and animal studies, standardized human dosing regimens, bioavailability, and toxicity profiles remain poorly defined. In the future, multicenter, prospective clinical trials will be essential to strengthen the clinical translation of these preclinical findings. Lack of experiments investigating on the long-term effect and safety of the treatments also leads to concerns. Most novel therapeutic approaches, including autophagy inducers, natural compounds (e.g., resveratrol, icariin), immunomodulators, and pro-angiogenic agents, remain at the conceptual and investigational stages. Long-term studies are scarce, and critical issues such as optimal dosing, off-target effects, and sustained efficacy remain largely unexplored.

## Conclusion

Despite extensive research, fully identifying the contributing factors and unraveling their complex interplay in SONFH remains a significant challenge. Current studies often focus on isolated aspects of the disease, which hampers the integration of findings into a cohesive understanding of SONFH pathogenesis. Additionally, many promising outcomes, particularly from bioinformatics studies, still require further validation. Integrating multi-omics approaches and confirming bioinformatics predictions are essential steps toward developing more effective and personalized treatments for this debilitating condition.
